# Supplementary material for: Fetoscopic endoluminal tracheal occlusion with Smart-TO balloon: Study protocol to evaluate effectiveness and safety of non-invasive removal
Source: PLoS One. 2023 Mar 13;18(3):e0273878. doi: 10.1371/journal.pone.0273878 (PMC10010565; doi:10.1371/journal.pone.0273878)

CLINICAL TRIAL PROTOCOL

**Non-invasive removal of the Smart Tracheal Occlusion Device for Fetal Congenital Diaphragmatic Hernia:**

**A Feasibility Study**

**Protocol Acronym/short title**: **Smart Removal**

**Version number:** v4 **– Date**  30/06/2021

**EudraCT Nbr:** Not applicable

**Internal ref. nbr: S65423**

**Sponsor**

University Hospitals Leuven (UZ Leuven)

Herestraat 49, B-3000 Leuven

**Coordinating Investigator**

Jan Deprest

List of Participating Sites

*(as applicable)*

| **List Of Participating Sites** | **Principal Investigator** |
| --- | --- |
| UZ Leuven, Herestraat 49, B-3000 Leuven | Jan Deprest |

SIGNATURES

**Title:** Smart Tracheal Occlusion for Congenital Diaphragmatic Hernia: A Feasibility Study

**Protocol:** SMART-Removal

The undersigned confirm that the above referenced protocol has been acknowledged and accepted, and agree to conduct the Trial in compliance with the approved protocol, and will adhere to: the principles outlined in the requirements for the conduct of clinical trials in the EU as provided for in Directive 2001/20/EC or the EU Clinical Trial Regulation 536/2014 (as soon as in effect) and any subsequent amendments thereto, the ICH guidelines, the most recent version of the Declaration of Helsinki, the Belgian law of May 7^th^ 2004 regarding experiments on the human person (as amended) or the Belgian law of May 7^th^ 2017 related to clinical trials on medicinal products for human use (as soon as in effect), the EU General Data Protection Regulation 2016/679 (GDPR), relevant Belgian laws implementing the GDPR, the Belgian Law of August 22^nd^ 2002 on patient rights, and any other regulatory requirements and Standard Operating Procedures (SOPs), as applicable.

The undersigned agree not to disclose the confidential information contained in this document for any purpose other than the evaluation or conduct of the Trial, without prior written consent of the Sponsor.

The undersigned also commit to making the findings of the Trial publicly available through publication and/or other dissemination tools, in accordance with this protocol and applicable regulations, without any unnecessary delay and to provide an honest, accurate and transparent account of the Trial; and to explain any discrepancies or deviations from the approved Trial protocol.

**Coordinating Investigator**

7-4-2021

Jan Deprest, MD, PhD, FRCOG Signature Date

**Principal Investigator (Participating Site)** *(in case of monocentric Trial, the Principal Investigator is the same as the Coordinating Investigator)*

7-4-2021

Jan Deprest, MD, PhD, FRCOG Signature Date

Table of Contents

[2.1 Trial objectives 15](#_Toc72499408)

[2.2 Primary Endpoints 15](#_Toc72499409)

[2.3 Secondary Endpoints 15](#_Toc72499410)

[2.4 Trial Design 16](#_Toc72499411)

[2.5 Expected Duration of the Trial 16](#_Toc72499412)

[3.1 Inclusion criteria 16](#_Toc72499413)

[3.2 Exclusion criteria 16](#_Toc72499414)

[4.1 Participant consent and withdrawal of consent 20](#_Toc72499415)

[4.2 Selection of Participants / Recruitment 20](#_Toc72499416)

[4.3 Randomization Procedure / Blinding (if applicable) 20](#_Toc72499417)

[4.4 Unblinding 20](#_Toc72499418)

[4.5 Premature discontinuation of Trial treatment 20](#_Toc72499419)

[5.1 Investigational Medical device 21](#_Toc72499420)

[5.1.1 Identification of the medical device 21](#_Toc72499421)

[5.1.2 Components of the medical device 21](#_Toc72499422)

[5.1.3 Description of materials, contact time, and tissues in contact 22](#_Toc72499423)

[5.1.4 Intended purpose of the device 22](#_Toc72499424)

[5.1.5 Summary of training and expertise required to use the device 22](#_Toc72499425)

[5.1.6 Standard operation procedure 22](#_Toc72499426)

[5.1.7 Precautions and contraindications 23](#_Toc72499427)

[5.2 IMD Accountability 23](#_Toc72499428)

[5.3 Concomitant / Prohibited Medication / Treatment 24](#_Toc72499429)

[5.4 Rescue Treatment 24](#_Toc72499430)

[6.1 Definitions 25](#_Toc72499431)

[6.1.1. Adverse Event (AE) 25](#_Toc72499432)

[6.1.2 Serious Adverse Event (SAE) 25](#_Toc72499433)

[6.1.3 Adverse Device Effect (ADE) 25](#_Toc72499434)

[6.1.4 Serious Adverse Device Effect (SADE) 26](#_Toc72499435)

[6.1.5 Device Deficiency (DD) 26](#_Toc72499436)

[6.1.6 Adverse Events of Special Interest 26](#_Toc72499437)

[6.2 Adverse Events that do not require reporting 26](#_Toc72499438)

[6.3 Recording and reporting of Adverse Events 26](#_Toc72499439)

[6.3.1 Assessment 27](#_Toc72499440)

[6.3.2 Timelines for reporting 28](#_Toc72499441)

[6.3.3 Follow-up 28](#_Toc72499442)

[6.3.4 Pregnancy 29](#_Toc72499443)

[6.3.5 Death 29](#_Toc72499444)

[6.4 Recording and reporting of Device Deficiencies 29](#_Toc72499445)

[6.5 Reporting requirements to Ethics Committee’s (EC’s) and Competent Authorities (CA’s) 29](#_Toc72499446)

[6.5.1 Sponsor’s reporting of Serious Adverse Events and Device Deficiencies 29](#_Toc72499447)

[6.5.2 Annual reporting 30](#_Toc72499448)

[6.5.3 Overview reporting requirements 30](#_Toc72499449)

[6.6 Data Safety Monitoring Board (DSMB) 30](#_Toc72499450)

[7.1 Sample Size Determination 31](#_Toc72499451)

[7.2 Statistical Analysis 31](#_Toc72499452)

[7.2.1 Analysis 31](#_Toc72499453)

[7.2.2 Other Analysis 32](#_Toc72499454)

[7.3 Interim Analysis and Final Database Lock 32](#_Toc72499455)

[8.1 Data Collection Tools and Source Document Identification 32](#_Toc72499456)

[8.1.1 Operational aspects 32](#_Toc72499457)

[8.1.2 Legal requirements 33](#_Toc72499458)

[8.2 Audits and Inspections 34](#_Toc72499459)

[8.3 Monitoring 34](#_Toc72499460)

[8.4 Archiving 34](#_Toc72499461)

[9.1 Ethics Committee (EC) review & reports 34](#_Toc72499462)

[9.2 Peer review 35](#_Toc72499463)

[9.3 Regulatory Compliance 35](#_Toc72499464)

[9.4 Protocol / GCP compliance 35](#_Toc72499465)

[9.5 Data protection and participant confidentiality 35](#_Toc72499466)

[9.6 Insurance 36](#_Toc72499467)

[9.7 Amendments 36](#_Toc72499468)

[9.8 Post-Trial activities 36](#_Toc72499469)

List Of Abbreviations

| **Abbreviation** | **Definition** |
| --- | --- |
| (e)CRF | (electronic) Case Report Form |
| AE | Adverse Event |
| AESI | Adverse Event of Special Interest |
| APR | Annual Progress Report |
| ASR | Annual Safety Report |
| AR | Adverse Reaction |
| CA | Competent Authority |
| CI | Coordinating Investigator |
| CIOMS | Council for International Organizations of Medical Sciences |
| CM | Concomitant Medication |
| CSR | Clinical Study Report |
| CTP | Clinical Trial Protocol |
| DMP | Data Management Plan |
| DPA | Data Processing Annex |
| DTA | Data Transfer Agreement |
| DSMB | Data Safety Monitoring Board |
| DSUR | Development Safety Update Report |
| EC | Ethics Committee |
| EU | European Union |
| ECG | Electrocardiogram |
| EoT | End of Trial |
| FPFV | First Patient First Visit |
| GCP | Good Clinical Practice (latest version of ICH E6) |
| GDPR | General Data Protection Regulation |
| IB | Investigator’s Brochure |
| ICF | Informed Consent Form |
| ICH | International Conference on Harmonisation |
| IMP | Investigational Medicinal Product |
| ISF | Investigator Site File |
| JCI | Joint Commission International |
| LPLV | Last Patient Last Visit |
| MAH | Marketing Authorisation Holder |
| MP | Monitoring Plan |
| PI | Principal Investigator (Participating Site) |
| PRO | Patient Reported Outcome |
| SAE | Serious Adverse Event |
| SAP | Statistical Analysis Plan |
| SAR | Serious Adverse Reaction |
| SOP | Standard Operating Procedure |
| SmPC | Summary of Product Characteristics |
| SUSAR | Suspected Unexpected Serious Adverse Reaction |
| TMF | Trial Master File |
| CDH | Congenital diaphragmatic hernia |
| FETO | Fetoscopic Endoluminal Tracheal Occlusion |
| TO | Tracheal Occlusion |
| US | Ultrasound |
| MRI | Magnetic Resonance Image |
| o/e LHR | Observed-to-expected lung-to-head-ratio |
| PROM | Premature Rupture of Membranes |

FUNDING AND SUPPORT

| **Funder** | **Type of Financial or Non-Financial Support** |
| --- | --- |
| There is no funder | There is no funding |
|  |  |

ROLES AND RESPONSIBILITIES

The Principle Investigator (PI) is responsible for the conduct of the Trial at his Participating Site, and for protecting the rights, safety and well-being of the Trial participants. As such the PI must ensure adequate supervision of the Trial conduct at the Participating Site. If any tasks are delegated, the PI will maintain a log of appropriately qualified persons to whom he/she has delegated specified Trial-related duties. The PI will ensure that adequate training is provided and documented for all Trial staff, prior to conducting assigned Trial-related activities.

It is the Coordinating Investigator's (CI’s) responsibility to supervise the general conduct (e.g. Trial progress, communication, protocol training and support of the participating sites, annual reporting to the Ethics Committee (EC), end of Trial notification(s) and results reporting…) of the Trial. The CI fulfils both Investigator and Sponsor responsibilities, as outlined in International Conference on Harmonisation – Good Clinical Practice (ICH-GCP) E6(R2) and applicable regulations.

PI and CI shall each be referred to as «Investigator(s)».

Trial Synopsis

| Title of clinical Trial («Trial») | Smart Tracheal Occlusion for Congenital Diaphragmatic Hernia: A Feasibility Study |
| --- | --- |
| Protocol Short Title Acronym | SMART-Removal |
| Trial Phase (I, II, III, IV) | Feasibility |
| Sponsor name | University Hospitals Leuven (UZ Leuven)> |
| Coordinating Investigator | Jan Deprest |
| Contact Address CI | Herestraat 49, 3000 Leuven, Belgium |
| Contact Email CI | [jan.deprest@uzleuven.be](mailto:jan.deprest@uzleuven.be) |
| Contact Phone CI | +32 16 34 42 15 |
| EudraCT number | Not applicable |
| Other public database nbr | Not applicable |
| Principal Investigators and Participating Sites | Jan Deprest (UZ Leuven) |
| Medical condition or disease under investigation | Congenital diaphragmatic hernia (Orphan code: 2140) |
| Trial rationale | To demonstrate the ability to prenatally deflate the Smart-TO balloon by the magnetic fringe field generated by an MRI scanner, and its subsequent expulsion out of the fetal airways |
| Primary objective | To demonstrate the ability to prenatally deflate the Smart-TO balloon by the magnetic fringe field generated by an MRI scanner |
| Secondary objective(s) | To evaluate:   1. balloon expulsion from the fetal airways after deflation. 2. spontaneous deflation of the balloon. 3. any adverse events related to the balloon. 4. lung growth. |
| Trial Design | Non-comparative monocentric interventional study. |
| Endpoints | **Primary:**   - Deflation rate after MRI exposure, assessed through ultrasound (US) immediately after MRI exposure   **Secondary:**  Prenatal:   - Spontaneous balloon deflation prior to MRI exposure, assessed through ultrasound (US) - Lung growth assessed by US two weeks after balloon insertion. - Localisation of the balloon within 24 hours following deflation by MRI - Airway patency as on MRI following deflation - Gestational age at membrane rupture   Postnatal:   - Gestational age at delivery - Balloon expulsion from the fetal airways - Location of the balloon after delivery   Neonatal:  Tracheal diameter on first postnatal chest X-ray  At discharge from the neonatal unit:   - Assessment for any local side effects of the balloon (signs or symptoms of tracheomegaly and /or tracheomalacia) - Survival   Adverse events:   - Any event or observation that may indicate an adverse event, either in the mother or the fetus or newborn, at whatever time point between insertion and discharge from the neonatal unit, and whenever possible indicate the potential relationship with the use of the Smart TO |
| Sample Size | Net number required based on power calculation: 23  + expected loss rate: 7.8% (n=2) due to the need for removal on placental circulation or spontaneous balloon deflation  Total number of patients to be recruited: 25 |
| Investigational medical device | Smart-TO balloon and installation system  Reference number: 111260-000000  Manufacturer: BS-Medical Tech Industry, Niederroedern, France  Class: IIB  No CE label at this stage - advise of CMM has been requested |
| Active comparator product(s) | Not applicable |
| Maximum duration of treatment and Follow Up of a Participant | 49 days |
| Maximum duration of entire Trial | 60 months |
| Date anticipated First Patient First Visit (FPFV) | Cannot be anticipated at this moment: depends on FAGG approval |
| Date anticipated Last Patient Last Visit (LPLV) | + 60 months |
| Third parties | BSM-TI, manufacturer of the device |

Trial Flowchart

Schedule of Events – Trial specific Procedures / Assessments

|  | Selection visit | FETO  (27,0 – 31,6) | Day 1 post FETO | Weekly follow-up visit | Unplug  (34,0 – 34,6) or earlier if required | Within 24 hours after Unplug | Weekly  follow-up visit | Delivery and neonatal management |
| --- | --- | --- | --- | --- | --- | --- | --- | --- |
| Informed consent^1^ |  |  |  |  |  |  |  |  |
| Eligibility assessment |  |  |  |  |  |  |  |  |
| Demographics |  |  |  |  |  |  |  |  |
| Medical, surgical history |  |  |  |  |  |  |  |  |
| Fetal US^2^ |  |  |  |  |  |  |  |  |
| Hospitalization |  |  |  |  |  |  |  |  |
| Placement of Smart-TO balloon |  |  |  |  |  |  |  |  |
| Reporting of side effects |  |  |  |  |  |  |  |  |
| MRI magnetic field exposure |  |  |  |  |  |  |  |  |
| MRI (Image adquisition) |  |  |  |  |  |  |  |  |
| Management following current standardized guideliness[1] |  |  |  |  |  |  |  |  |
| X-ray newborn |  |  |  |  |  |  |  |  |
| Postpartum ultrasound uterus in mother if indicated |  |  |  |  |  |  |  |  |

1: *Informed Consent be obtained prior to performing any other Trial-related procedures; 2:* Ultrasound (US) (Fetal heart rate, presentation, amniotic fluid, cervical length, and observed-to-expected-lung-to-head-ratio (o/e LHR)); Green indicates standard of care; Blue indicates additional interventions specific for the trial.

# Background, Rationale and Risk Assessment

**Background**

**Congenital diaphragmatic hernia (CDH)** is a birth defect characterized by impaired closure of the diaphragm. This enables abdominal viscera to herniate into the thoracic cavity, leading to hypoplastic lungs and impaired lung vasculature[2]. Fetal lung growth may be stimulated by Fetoscopic Endoluminal Tracheal Occlusion (FETO)[3]. Compared to historical data, FETO increases survival in severe left CDH from 24% to 49% (p<0.001)[4] and in severe right CDH from 15% to 41% (p=0.014)[5]. FETO may also reduce early neonatal morbidity[6]. For left sided CDH, there is now level I evidence that it significantly improves survival in severe cases; the effect in moderate cases is much less.

Despite its benefits, the current procedure has many disadvantages. First, there is the need for prenatal reversal of the balloon, which is invasive. In utero balloon removal re-establishes airway patency. This can be done electively at 34 weeks, or earlier if required, but preferentially at least 24 hours before birth[6]. “Unplugging” requires a second procedure and a specialist team familiar with the procedure and that is available at all times[7]. In the largest series published thus far, 28% of balloon removals were in an emergency setting. The only neonatal deaths were caused by complications when balloon reversal was attempted in centers without experience or that were unprepared[7]. In utero reversal of the occlusion is also an invasive procedure, because it requires ultrasound guided puncture or fetoscopy. The second procedure adds both to the fetal and maternal risks:

- Balloon removal is a difficult procedure which needs expertise[7].
- Prenatal balloon removal procedure can fail in 3.4% even in experienced centers[7].
- Balloon removal is not feasible *in utero* in 12.6 to 24.7% of the cases[7].
- Balloon removal is performed in an emergency setting in 28% of the cases[7].
- Balloon removal can lead to neonatal death in none well-trained centers[7].
- *In utero* balloon removal may induce delivery within 1 week in 25% of cases[7].
- Patients are requested to stay close to a FETO center for the entire duration of the tracheal occlusion, which is a a burden on the family, limiting the acceptability of FETO[7].

**Smart-TO balloon for FETO**

The University of Strasbourg, in partnership with BSMTI, Niederroedern, France, developed an alternative occlusion device, referred to as "Smart-TO". Compared to the Goldbal2® balloon, which is typically used, the Smart-TO balloon has the same dimensions in its inflated state, and is made of the same material (latex). Around the balloon neck there is a metallic cylinder and inside a magnetic ball, which acts as a valve. Deflation occurs under a strong magnetic field, as generated by a clinical MRI machine, details provided below. For that it is sufficient for the pregnant woman to walk around a clinical MRI machine. This enables non-invasive, externally controlled balloon deflation and avoids a second surgery for airway reestablishment. The empty balloon is expelled from the airways by the accumulated fetal lung secretions below the occlusion and by fetal respiratory movements (as currently the case with the standard Goldbal2 balloon following ultrasound (US) guided puncture).

**Preclinical studies on the Smart-TO balloon**

The Smart-TO balloon is the result of several years of research and development within a consortium of the following institutions: Strasbourg University Hospital, University of Strasbourg, INSERM Unit 1121 "Biomaterials and Bioengineering", Simian Laboratory Europe, Institut Hospitalo-Université de Strasbourg, Institute for Research Against Cancers of the Digestive System, SATT-Conectus Alsace, BS-MTI and, for the more recent preclinical experimentation, the KU Leuven.

The following tests have already been completed the following test have been completed using an identical version (V0) of the Smart-TO device intended for clinical purposes:

In-vitro:

- Permeability, occlusion, and deflation in a simulated laboratory environment [8] (Sananès Ph.D. Thesis, 2017, University of Strasbourg), and with a mannequin in a simulated "in-utero" environment, with the fetus in different fetal positions and the mother in different positions and heights. Of relevance to this study is that balloon deflation was successfully achieved regardless of the fetal position and the exact level of the simulated uterus from ground level (Basurto et al., KU Leuven, UOG, 2020)[9].

In-vivo:

- Balloon insertion, persisting occlusion until exposure to a magnetic field, and spontaneous expulsion of the Smart-TO balloon was demonstrated in a limited number of non-human primates (University of Strasbourg)[8]
- Occlusiveness and acute tracheal side effects in fetal lambs. In that study, the Smart-TO balloon had similar effects as the ones induced by the standard Goldbal2^®^ system, i.e., it induced similar lung growth and superficial tracheal changes [10].
- Efficacy and safety in fetal lams with CDH. In that experiment, the occlusion was non-invasively reversed and the Smart-TO balloon expelled from the airways. Also the occlusion rescued pulmonary hypoplasia with minimal tracheal changes[9].

**Rationale and clinical goal**

The above preclinical data justify translation to clinical practice. We propose a first-in-woman study wherein we aim to demonstrate to reverse the occlusion induced by the Smart-TO balloon by the magnetic fringe field generated by an MRI scanner.

# Trial Objectives and Design

## Trial objectives

The main objective is to demonstrate the ability to prenatally deflate the Smart-TO balloon by the magnetic fringe field generated by an MRI scanner.

Secondary objectives are to evaluate:

- balloon expulsion from the fetal airways after deflation.
- spontaneous deflation of the balloon.
- any adverse events related to the balloon.
- lung growth.

## Primary Endpoints

The primary endpoint is the deflation rate after MRI exposure, assessed through US immediately after MRI exposure. When inflated, the balloon is easily visible on US as an anechoic structure. Balloon deflation will be indicated by visualization of the balloon on US before MRI-exposure and its disappearance immediately after MRI exposure. Two experienced operators will perform this US examination. In a previous study by our group, US was 100% accurate in the determination of airway patency following ultrasound guided balloon puncture (n=63).

## Secondary Endpoints

Secondary endpoints are:

Prenatal:

- Spontaneous balloon deflation prior to MRI exposure, as diagnosed through US. Although a rare event, this has been reported with the standard Goldbal2^®^ balloon in 3.1%[11].
- Lung growth assessed by US prior to deflation, measured by change in the observed-to-expected lung-to-head-ratio (o/e LHR) before balloon insertion and two weeks after FETO[12, 13].
- Localisation of the balloon within 24 hours following deflation by MRI visualization.
- Airway patency as on MRI following deflation
- Gestational age at membrane rupture

Postnatal:

- Gestational age at delivery
- Localisation of the balloon either by (1) direct visualization within the amniotic fluid, membranes or placenta, (2) postnatal chest X-ray of the newborn, and (3) ultrasound of the postpartum uterus.

Neonatal:

- Tracheal diameter on first postnatal chest X-ray at:[14]
  - - the level of entry into the chest (mm)
    - 1 cm above the carina (mm)
    - at mid-distance between these sites (mm).

At discharge from the neonatal unit:

- Assessment for any local side effects of the balloon (signs or symptoms of tracheomegaly and /or tracheomalacia)
- Survival

Adverse events:

- Any event or observation that may indicate an adverse event, either in the mother or the fetus or newborn, at whatever time point between insertion and discharge from the neonatal unit, and whenever possible indicate the potential relationship with the use of the Smart-TO device.

## Trial Design

This is a non-comparative monocentric interventional study. All included cases will undergo FETO with the Smart-TO balloon.

## Expected Duration of the Trial

- Duration of participation (treatment + follow-up): 6 months
- Estimated total duration: 60 months. Based on the number of FETO procedures performed in the last five years (n=34).

# Trial Population / Eligibility Criteria

## Inclusion criteria

Participants eligible for inclusion in this Trial must meet **all** of the following criteria:

1. Women carrying a fetus with isolated CDH eligible for FETO, i.e. with severe or moderate hypoplasia:
   1. Left sided CDH with severe (i.e. quotient of observed-to-expected- lung-to-head ratio under 25%) or moderate pulmonary hypoplasia (observed-to-expected- lung-to-head ratios of 25 to 34.9% (any liver position), or 35 to 44.9% with intrathoracic liver herniation)[12, 13].
   2. Right sided CDH with severe pulmonary hypoplasia (quotient of observed-to-expected- lung-to-head ratio under 50%)[5].
2. Voluntary written informed consent of the participant or their legally authorized representative has been obtained prior to any screening procedures
3. Maternal age of 18 years or older
4. Single pregnancy
5. Signed informed consent
6. Consent to have an ultrasound of the uterus after delivery when indicated
7. Postnatal management center agrees to participate

All participants that are considered for Trial participation per the above criteria will be documented on the Screening Log, including Screen Failures.

## Exclusion criteria

Participants eligible for this Trial must **not** meet any of the following criteria:

1. History of allergy to latex
2. Contraindication to fetoscopy and FETO (maternal diseases or technical limitations making prenatal surgery hazardous or impossible)
3. Premature Membrane Rupture (PROM)
4. Multiple pregnancy
5. Maternal age less than 18 years
6. Refusal to stay close to the UZ Leuven until balloon removal
7. Any disorder, which in the Investigator’s opinion might jeopardise the participant’s safety or compliance with the protocol
8. Any prior or concomitant treatment(s) that might jeopardise the participant’s safety or that would compromise the integrity of the Trial
9. Participation in another interventional trial with an investigational medicinal product (IMP) or device

Participants who meet one or more of the above exclusion criteria **must not proceed** to be enrolled in the Trial and will be identified on the Screening Log as Screen Failure.

# Trial Procedures

* indicates interventions that are specific for the trial apart of the current standard clinical practice in patients undergoing FETO.

**Selection visit**

The selection visit will occur at the latest one day before the balloon placement.

The inclusion criteria will be checked as per current clinical practice of CDH patients; therefore, this visit will be the same for all patients carrying a CDH fetus.

After consultation, the patient will be counselled by a specialist in fetal medicine. Consent will be gathered after at least 24 hours of reflection and after the physician has answered all of her questions.

**First study intervention**

Once the patient is included in the study and the date of FETO is scheduled, the following procedures will be performed:

Day before FETO:

- Preoperative blood test
- Anaesthesia consultation
- Control US (fetal heart rate, presentation, amniotic fluid assessment, cervical length measurement, and o/e LHR).
- Hospitalization on the day of balloon placement or the evening before the surgery, according to the patient's preference and availability of the delivery unit.

Day of FETO:

- FETO with the Smart-TO balloon*.

Day 1 after FETO

- Control US (fetal heart rate, balloon position, presentation, amniotic fluid assessment, cervical length measurement, and o/e LHR).
- In the absence of complications, the patient will be discharged from the hospital with instructions to come back in case of PROM, fever, contractions, or any other complication. The patient will be requested to remain in the Leuven region for the tracheal occlusion duration to ensure an optimal follow-up and take care of possible PROM or preterm delivery.

**Follow-up visits**

Follow up between day one after FETO and balloon removal:

- Weekly US to assess fetal vitality, presentation, amniotic fluid, cervical length, balloon position, and o/e LHR.
- Clinical and serological follow-up of pregnancy.

**Second study intervention**

Balloon deflation (unplug) will be scheduled between 34,0 and 34,6 weeks of GA or earlier if necessary. In the unlikely event of a patient with imminent signs of delivery, balloon removal will be done on placental circulation or postnatally. This will ensure the patient's safety.

We therefore foresee two different scenarios in the present study:

1. Removal in case of non-imminent delivery.

This will include elective balloon deflation at 34 weeks of gestation and earlier than planned balloon deflations (due to any clinical circumstance) without imminent signs of delivery. We expect this to be the case in 95% of the cases (based on previous data)[11].

- Before MRI exposure, a control US will be performed by two experienced sonographers (fetal heart rate, balloon position, presentation, amniotic fluid assessment, and cervical length measurement and o/e LHR).
- Smart-TO balloon deflation protocol (described in section 5.1.9).*
- Control US performed by two experienced sonographers to assess balloon deflation. In the case of deflation failure, a second and -if necessary- a third MRI exposure* will be attempted following US confirmation to ensure balloon deflation. In the case of balloon failure to deflate or any doubt about deflation, an MRI with image acquisition will be performed to (1) attempt deflation, and (2) assess the airway patency. In the case of failure to deflate, balloon removal will be done by fetoscopy, placental circulation of postnatally (depending on the clinical scenario). The patient will be informed of this possibility although the risk of this is extremely low according to all preclinical tests.

Within 24 hours after balloon deflation:

- An MRI (with image acquisition) will be performed to locate the deflated balloon and as a second safety measure to ensure the patency of the airways.
- The patient will be allowed to return to her tertiary centre of preference once the airways' patency is confirmed.

In current practice, when reversal of the occlusion is performed by puncture, the deflated balloon is expelled outside the airways by a spontaneous outflow of the fluid accumulated under the balloon and fluid exiting during fetal breathing movements. After balloon deflation (also with the current Goldbal2 balloon), the deflated balloon's fate is going to from the pharynx to either the gastrointestinal tract, followed by spontaneous expulsion, or to the amniotic fluid. From there, it should be expelled during delivery or after membrane rupture. Note that the Goldbal2 balloon currently used for tracheal occlusion also has a metallic ball component, and no adverse events have been ever reported.

In the case the Smart-TO balloon is ingested, one can consider this as a situation equivalent to the accidental ingestion of a magnetic object by small children. Such a situation is considered innocuous and expectantly managed[15, 16].


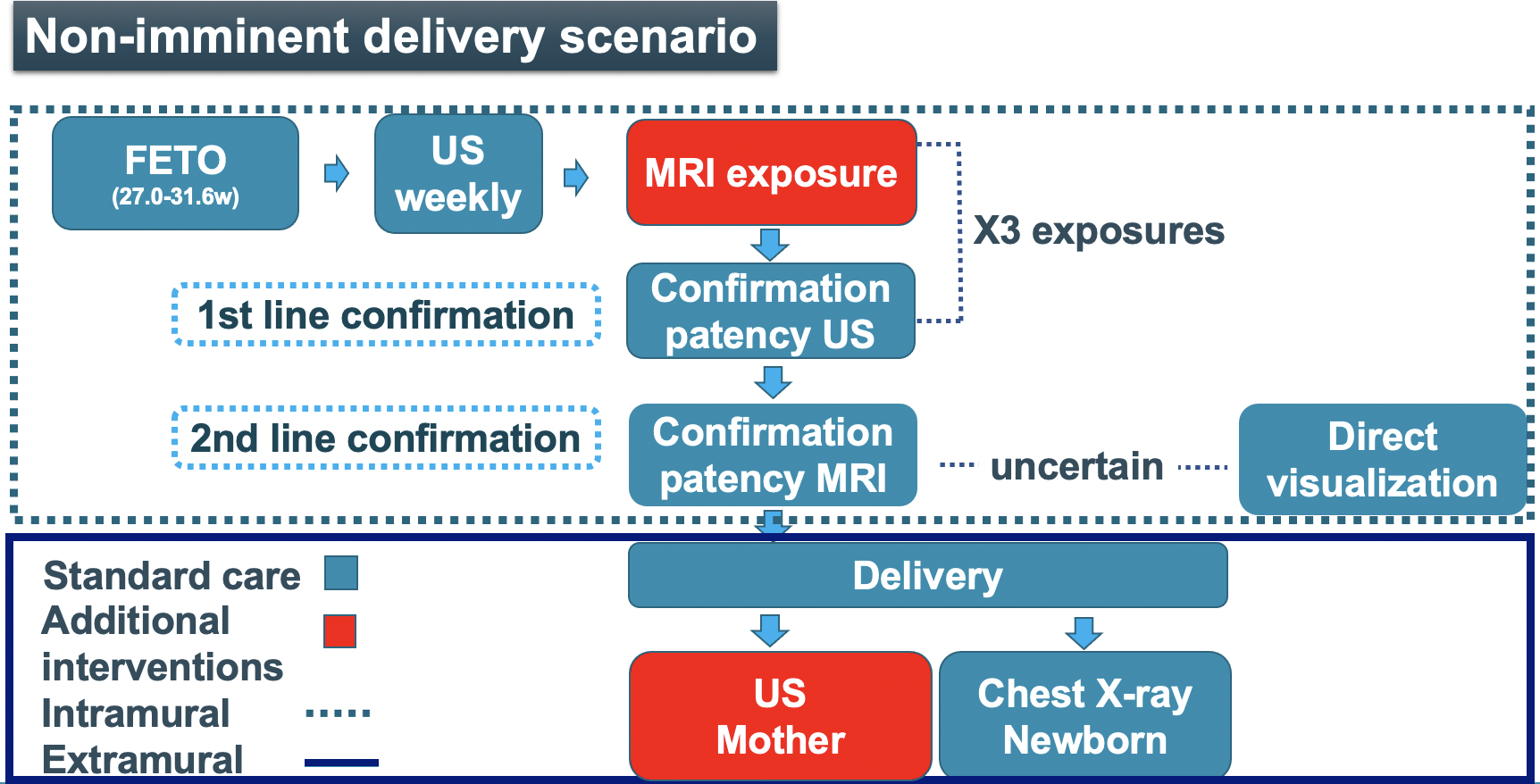


**Figure 1: Non-imminent delivery scenario.**

1. Removal in case of imminent delivery.

We will manage that situation as we currently clinically do with the standard balloon. This includes direct visualization at the time of removal, either by fetoscopy or tracheoscopy on placental circulation, or in an emergency, postnatally (Figure 2). We expect this scenario to happen in maximum 5% of the cases based on a multicentre study we coordinated [11]. In our hands that number is even lower.


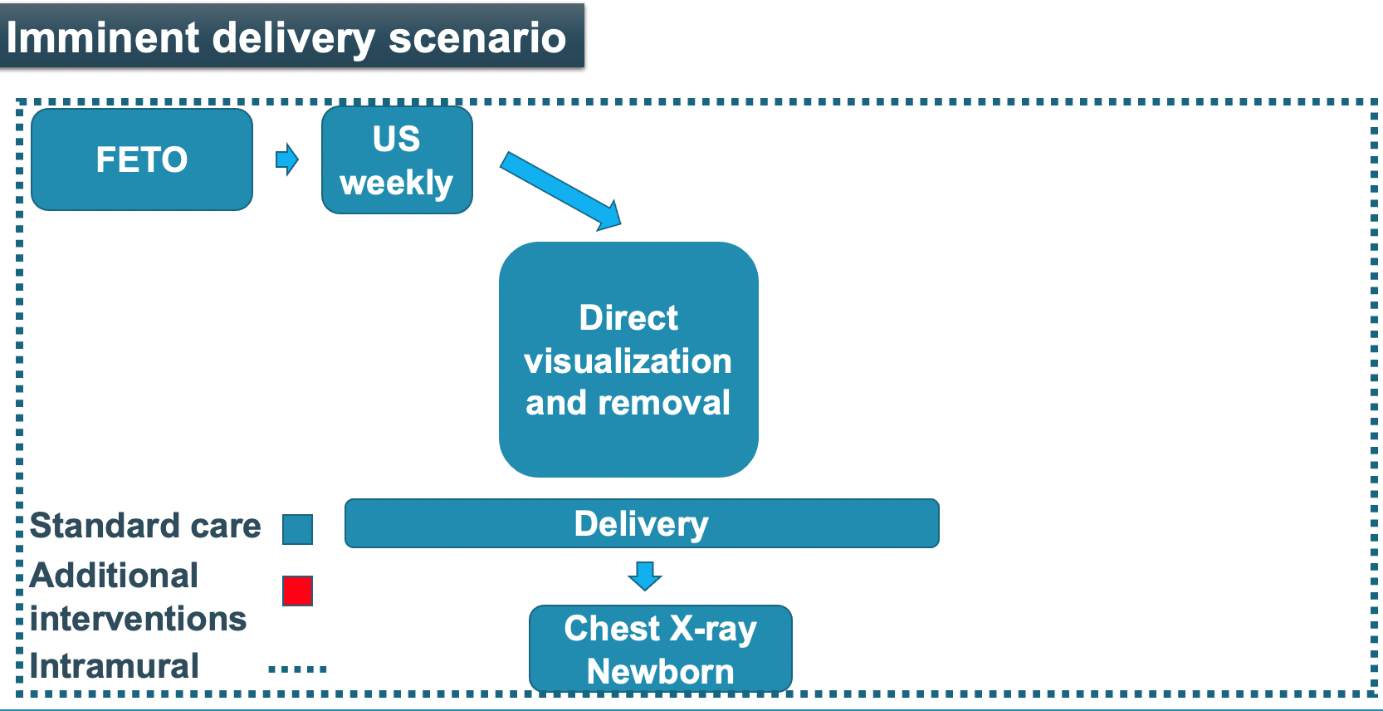


**Figure 2: Imminent delivery scenario.**

**Delivery**

- - Delivery does not differ from the standard care protocol for children with CDH[1].
  - In case of MRI deflation protocol, the following will be done to locate the balloon:

(1) Inspection and search of any amniotic fluid, membranes, and the placenta*

(2) Standard X-ray of the newborn, which is standard, to assess lungs, airways, intubation and location of the stomach and naso-gastric tube; on that the balloon may be visible e.g. in the stomach.

(3) An ultrasound of the postpartum uterus will be done to demonstrate the uterine cavity is empty.

**Neonatal management**

- The follow-up and mangement of the newborn will be according to the standardized management protocol of the CDH EURO Consortium[1].
- We will screen for the presence of tracheomegaly and/or tracheomalacia, which is part of the standard care in FETO patients.
- Data collection will be stopped at the moment the child is discharged from the hospital.

## Participant consent and withdrawal of consent

The Trial will be conducted only on the basis of prior informed consent by the Trial participants and/or their legally authorized representative(s). As such, no Trial-related procedures will be conducted prior to obtaining written informed consent from potential Trial participants.

The process for obtaining and documenting initial and continued informed consent from potential Trial participants will be conducted in accordance with ICH-GCP E6(R2), applicable regulatory requirements and internal Standard Operating Procedures (SOPs).

All originally signed obtained Informed Consent Forms (ICFs) must be retained/archived in the Investigator Site File (ISF) at the Participating Site and must not be destroyed (even when a scanned copy is available) before expiration of the legal archiving term as defined in the protocol section entitled “Archiving”.

Participants may voluntarily withdraw consent to participate in the Trial for any reason at any time. The participant’s request to withdraw from the Trial must always be respected without prejudice or consequence to further treatment. Consent withdrawal will be documented in the participant’s medical record.

Trial data and samples collected before withdrawal can be used in the trial. No new trial data or samples will be collected after withdrawal of the participant.

## Selection of Participants / Recruitment

Recruitment of participants will be at the Fetal Medicine Unit (FMU). The selection visit takes place at least one day before the balloon placement.

## Randomization Procedure / Blinding (if applicable)

This is not a randomised trial; all eligible patients will undergo FETO with the Smart-TO balloon.

## Unblinding

Not applicable. Blinding will not be used since this is a non-comparative interventional trial.

## Premature discontinuation of Trial treatment

Participants may voluntarily discontinue from Trial treatment and/or prematurely end their participation in the Trial for any reason at any time. In such case the Investigator must make a reasonable effort to contact the participant (e.g. via telephone, e-mail, letter) in order to document the primary reason for this decision.

The Investigator may also decide at any time during the course of the Trial, to temporarily interrupt or permanently discontinue the Trial treatment if it is deemed that continuation would be detrimental to, or not in the best interest of the participant.

Similarly, the Sponsor, Ethics Committee or authorized regulatory authority can decide to halt or prematurely terminate the Trial when new information becomes available whereby the rights, safety and well-being of Trial participants can no longer be assured, when de integrity of the Trial has been compromised, or when the scientific value of the Trial becomes obsolete and/or unjustifiable.

Circumstances requiring premature treatment interruption or discontinuation of the Trial, include but are not limited to:

- Safety concerns related to investigational medical device or unacceptable intolerability
- Trial participation while in violation of the inclusion and/or exclusion criteria

In any such case of early Trial termination and/or treatment interruption/discontinuation, the Investigator will continue to closely monitor the participant’s condition and ensure adequate medical care and follow-up. All relevant adverse events identified will be reported as required to all relevant authorities.

For participants whose status is unclear because they fail to appear for Trial visits without stating an intention to discontinue or withdraw, the Investigator must make every effort to demonstrate “due diligence" by documenting in the source documents which steps have been taken to contact the participant to clarify their willingness and ability to continue their participation in the Trial (e.g. dates of telephone calls, registered letters, etc.).

A participant should not be considered lost to follow-up until due diligence has been completed.

# Trial Medical Device

| **Medical Device Name  (& company brand name)** | **IMP or non-IMP** | **Used within Indication? (Y or N)** | **Route of administration (po,sc,iv,…)** | **Dose/dosage and units** |
| --- | --- | --- | --- | --- |
| Smart-TO (BS-Medical Tech Industry) | Not applicable | Y | Insertion by fetoscopy (surgical procedure) | Not applicable |

## Investigational Medical device

### Identification of the medical device

- Name: Smart-TO balloon and installation system Model: V0
- Reference number: 111260-000000
- Manufacturer: BS-Medical Tech Industry, 2 rue de l'avenir, 67470 Niederroedern, France
- Sterilization: Ethylene Oxide (EO)
- Class of the device: IIB

The product is not CE marked at this stage of the study.

### Components of the medical device

The medical device is a set composed of several elements (Appendix 3, Figure 1):

- The Smart-TO balloon
- The delivery system (Ø1.3mm length 935mm)
- Deflation stylet Ø0.2mm length 937mm
- One Luer Lock syringe, 1 mL

The Smart-TO balloon is the part of the device placed within the fetal trachea (> 30 days). It comes in its peel-off bag. The balloon is equipped with a magnetic valve and has the dimensions of Ø7mm x 20mm after filling with 0.7ml of sterile saline, which are the same dimensions of the standard Golbal^®^ balloon used for FETO within the TOTAL trial.

The delivery system allows the balloon to be deployed in the fetal trachea. It is calibrated to pass through the operator channel of the fetoscope. It has the following dimensions: diameter 1.3 mm and length 935 mm. It is equipped with a Luer Lock connector on which the syringe is attached to fill the balloon.

The set comes with two deflation stylets that allow the "mechanical" opening of the balloon's magnetic valve (test procedure before balloon placement and deflation procedure in case of poor placement in the trachea before being released). Dimension are 0.2 mm in diameter and 937 mm in length.

Finally, the set includes a 1 mL Luer Lock syringe to fill the balloon with sterile saline.

The device is sterilized with ethylene oxide (EO) by the manufacturer. Validation of the sterilization cycle was carried out according to DIN EN ISO 11135: 2014.

### Description of materials, contact time, and tissues in contact

The entire device consists of biocompatible materials. All materials are commonly used in medical devices, except for the ball and the ring (valve elements). These elements are coated with a thin film of parylene to ensure biocompatibility. The main element of the balloon is medical-grade latex, which has undergone biocompatibility tests.

The contact time with the patient is greater than 30 days, which classifies the device as class IIB according to the directive 93/42 of the medical devices (OJ L 169, 12.7.1993, p. 1). The balloon is in contact with the fetal trachea and the amniotic and tracheal fluid. Following deflation by the magnetic fringe field of a 3 Tesla MRI scanner, the balloon is expected to be expelled to the amniotic cavity due to the accumulated fluid pressure below the balloon and the fetal respiratory movements (as per current clinical practice with the standard Goldbal^®^ balloon). The balloon will remain in the amniotic cavity until the delivery. At that moment, the deflated balloon should be expelled from the patient's body within the amniotic fluid, placenta, membranes, and by normal vaginal lochia post-delivery (as per current clinical practice when US-guided puncture is performed for the standard Goldbal^®^ balloon).

Regarding the delivery system, only the system's proximal end comes into contact with the fetal trachea and the amniotic fluid during the procedure. The rest of the system remains inside the fetoscope's working channel and may eventually come into contact with the patient's skin.

The deflation stylet may come into contact with the patient's skin during its use in the unlikely case that the recently positioned balloon needs to be replaced. This eventuality has never occurred in preclinical animal experiments, but it is still an option for the user as a safety measure.

### Intended purpose of the device

The Smart-TO device has been specifically designed for FETO as a treatment for CDH. The device is intended to be introduced through the operating channel of a fetoscope to allow the placement of the balloon in the fetal trachea (as in the current clinical procedure) [17]. (Appendix 3, Figure 2)

The instructions of use by the manufacturer are provided in Appendix 3, Figure3.

### Summary of training and expertise required to use the device

The user must be fully trained for FETO with the Goldbal® balloon.

According to conducted preclinical trials, the setup of the Smart-TO is easier than the Goldbal® device; therefore, it requires minimal training.

A user manual is provided with each set to become familiar with the device and its operation. Our group has undergone more than 100 preclinical balloon placements; hence, we have complete knowledge regarding the device's setup and operation.

### Standard operation procedure

#### FETO

According to the current standards, the FETO procedure will be performed as described in a recent publication from our group [17]. Briefly,

-The mother will undergo local or loco-regional anesthesia (depending on the patient's preference and fetal position).

-Fetal anaesthesia and analgesia will be achieved by intramuscular or umbilical cord injection of a combination of Fentanyl®, curare, and Atropine® under US guidance.

-The fetoscope and camera will be set up.

-The delivery system will be introduced in the fetoscope's operating channel, and the Smart-TO balloon will be mounted in the delivery system.

-The balloon will be tested (inflated with 0.7 mL of sterile saline solution and deflated with its proper stylet).

-A 10 F trocar will be inserted through the abdominal wall into the amniotic cavity.

-The fetoscope (Storz Ref 11540AA and 11540KC) will be introduced first into the amniotic cavity and then into the fetal oral cavity under direct visualization with the aid of US guidance.

-The balloon will be positioned between the carina and the vocal cords, will be filled with 0.7 mL sterile saline solution and will be detached by gentle traction of the delivery system.

-All instruments will be removed, and the skin sutured with a single stay suture of Vicryl 3/0.

#### Reestablishment of the fetal airways

Smart-TO balloon deflation protocol

The balloon can be deflated by de-activating the magnetic valve. In order to do so, the deflation protocol must be performed by exposure to an MRI machine (referred to as MRI) as follows (Appendix 3, Figure 4)

- The patient will be positioned in front of the MRI, the abdomen facing to the front of the tunnel of the machine.
- The patient will walk (or be strolled) around the machine while staying as close as possible to the machine.
- When approaching the rear of the tunnel, the patient will position herself in the centre facing the tunnel and make a short stop.
- Then she will continue to walk (or being strolled) around the MRI while staying as close as possible to the machine
- Once she has completed the turn she can leave the MRI room

Conventional reestablishment of the fetal airways

The balloon removal takes place in essentially the same preparation described above for balloon placement, with the same fetal and maternal medications. Any of the following modalities are avaibale:

- Balloon puncture using a 20-22G needle under US guidance.
- Fetoscopy
- In the event that in utero removal is not possible, we resort to tracheoscopic removal on placental circulation under locoregional anesthesia

### Precautions and contraindications

The patient will be followed according to the clinical protocol.

The waste must be treated according to the hospital standards for the treatment of disposable devices. There are no special precautions to take.

The product cannot be reused or re-sterilized.

The patient must not be exposed to an MRI machine throughout the time that the balloon is in place, which may trigger the balloon's deflation involuntarily and irreversibly. The simple fact of being subjected to the residual magnetic fringe field will cause the magnetic valve's opening.

Note: Weak magnetic fields encountered in everyday life do not cause deflation of the balloon. As a precaution, it is not recommended to go through airport security portals.

## IMD Accountability

Delegated study personnel within the hospital pharmacy of UZ Leuven will receive and dispense the Investigational Medical Device (IMD) used for the clinical trial according to the current legislation and subject to mandatory official approval for the use of non-CE-labeled medical devices.

Under no circumstances will an investigator of pharmacist allow the IMD to be used other than directed by the study protocol.

The hospital pharmacist will provide in the hospital information system (electronical prescription and stock management system) the functionality for accountability and traceability of the IMD.

The investigator is responsible to provide the necessary data to the hospital information system in order to guarantee accountability and traceability.

#### Presentation

The sets are packaged individually. Each external packaging will include, in addition to the regulatory notices applicable to the IMD, a counter-labeling which will dedicate them exclusively for research.

#### Conservation-storage

IMDs must be stored in a locked place, allowing the storage instructions specified on the packaging to be observed. Conditions for IMD storage: dry place at room temperature and away from light.

#### Supply

The supplies are managed by the partner company BS Medical Tech Industry.

Following pharmaceutical agreement of the center, a first supply request of eight kits will be issued by the sponsor.

The following replenishments will be requested by the hospital pharmacy on the basis of:

- a predefined threshold of 4 kits available in the center,

or

- anticipation of the expiry of units in stock in the center;

A restocking period of around 60 days should be anticipated in both cases.

The internal circuit of dispensations / endowments will guarantee the presence of at least one emergency IMD available before each intervention (in the event of a malfunction).

#### Use and traceability of IMDs

Three detachable stickers are present on the labeling of the IMD and must be fixed at least

- in a register stored by the PI

- scanned and saved in the patient medical file

#### Quality complains / batch recall

In the event of any anomaly / quality deviation, a defect follow-up sheet will be completed by the declarant and sent to Study Monitor and supplier for materiovigilance data.

In the event of a batch recall, the pharmacy will be informed by the promoter who will set up the corresponding procedure (quarantine, inventory of stocks on site, and replacement, etc.).

#### Return of IMDs

In the event of expiry, this will be logged on an agreed study accountability log before request for returns to the supplier.

Once the target number of participants is achieved and the last patient has completed her final study visit, the Study Monitor will conduct close-out procedures, which will include ensuring accountability of IMD.

## Concomitant / Prohibited Medication / Treatment

All treatments and examinations are allowed except for MRI exposure during the duration of tracheal occlusion with the Smart-TO balloon as it may cause unintentional balloon deflation.

## Rescue Treatment

- In the event malfunction of the Smart-TO device is detected during pre-insertion testing, the practitioner may resort to the standard plug technique using the Goldbal® device [17].
- In case the Smart-TO device fails to deflate after MRI exposure, the physician may resort to the standard technique to reestablish the fetal airways[17].

# Safety Reporting

## Definitions

The definitions and reporting requirements adopted in this Clinical Investigation Plan (CIP) are based on the Medical Device Regulation (EU) 2017/745 and the MDCG 2020-10/1 European guideline.

### Adverse Event (AE)

An AE is any untoward medical occurrence, unintended disease or injury or any untoward clinical signs (including an abnormal laboratory finding) in subjects, users or other persons, in the context of a clinical investigation, whether or not related to the investigational medical device.

Note:

a. This definition includes events related that are anticipated as well as unanticipated events

b. This definition includes events occurring in the context of a clinical investigation related to the investigational device, the comparator or the procedures involved.

Since this is a feasibility study, all AE's occurring from treatment initiation until discharge of the patient and the neonate will be reported.

### Serious Adverse Event (SAE)

A SAE is any adverse event that led to any of the following:

a) death,

b) serious deterioration in health of the subject, that resulted in any of the following:

- life-threatening illness or injury,

- permanent impairment of a body structure or a body function,

- hospitalisation or prolongation of patient hospitalisation,

- medical or surgical intervention to prevent life threatening illness or injury or permanent impairment to a body structure or a body function,

- chronic disease,

c) fetal distress, fetal death or a congenital physical or mental impairment or birth defect.

The following are events will still be reported as SAE but are part of the natural history of CDH or are known potential complications of FETO:

Related to CDH

- **Fetal risks:**
- Hydrops
- Intrauterine fetal death, for as long as<5% of cases, because CDH fetuses are at increased risk for intrauterine fetal death. The average incidence of this complication is 3-5%[18].
- Neonatal death: CDH is associated with an overall mortality of about 30% [18]; in cases eligible for fetal intervention the mortality with expected management is around 50-80%.

Related to FETO

- Tracheal perforation by fetoscopy during balloon insertion.
- **Maternal risks:**
- Chorio-amnionitis.
- Placental abruption.
- Preterm rupture of membranes may prompt readmission of the patient.
- Preterm labour induced by the intervention or by subsequent preterm rupture of membranes

### Adverse Device Effect (ADE)

An ADE is an adverse event related to the use of an investigational medical device.

### Serious Adverse Device Effect (SADE)

A SADE is an adverse device effect that has resulted in any of the consequences characteristic of a serious adverse event.

An Unanticipated Serious Adverse Device Effect is an effect which by its nature, incidence, severity or outcome has not been identified in the current risk assessment.

### Device Deficiency (DD)

A DD is any in the identity, quality, durability, reliability, safety or performance of an investigational device, including malfunction, use errors or inadequacy in information supplied by the manufacturer.

### Adverse Events of Special Interest

The following events should be reported within the same timelines as SAEs:

- - Malfunction of the medical device (e.g., inability to deflate, inability to be expelled from the fetal airways, spontaneous deflation, inability to remove the balloon leading to birth asphyxia).
- Tracheomalacia (known side effects of FETO in about 3%).

## Adverse Events that do not require reporting

In general, the following should not be reported as AEs:

- Pre-existing conditions, including those found as a result of screening (these should be reported as medical history or concomitant illness.
- Pre-planned procedures, unless the condition for which the procedure was planned has worsened from the first trial-related activity after the subject has signed the informed consent.

The following events are commonly observed after FETO and are therefore not considered as adverse events for the purpose of the trial:

- Amniotic fluid leakage into the maternal abdomen, leading to peritoneal irritation.
- Minor bleeding from the uterine entry site

Although these events should not be reported to the Sponsor, these should be recorded in the patient’s medical notes according to routine practice.

The following events not to be considered as SAEs are:

- Pre-planned hospitalisations unless the condition for which the hospitalisation was planned has worsened from the first trial-related activity after the subject has signed the informed consent.
- Hospitalisation as part of a standard procedure for protocol therapy administration. However, hospitalisation or prolonged hospitalisation for a complication of therapy administration will be reported as an SAE.
- Hospitalisation or prolongation of hospitalisation for technical, practical, or social reasons, in absence of an AE.

## Recording and reporting of Adverse Events

Investigators will seek information on AEs during each patient contact. All events, whether reported by the patient or noted by trial staff, will be recorded in the patient’s medical record and in the (e)CRF within a reasonable time after becoming aware. If available, the diagnosis should be reported on the AE form, rather than the individual signs or symptoms. If no diagnosis is available, the Investigator should record each sign and symptom as individual AEs using separate AE forms.

The following minimum information should be recorded for each AE:

- AE description

- start and stop date of the AE

- severity

- seriousness

- causality assessment to the Investigational Medical Device (IMD) and/or study procedures

- outcome

### Assessment

All AEs must be evaluated by an Investigator as to:

- **Seriousness:** whether the AE is an SAE. See above for the seriousness criteria.
- **Severity:**
  - Severity must be evaluated by an Investigator according to the following definitions:
    - *Mild* – no or transient symptoms, no interference with the subject’s daily activities
    - *Moderate* – marked symptoms, moderate interference with the subject’s daily activities
    - *Severe* – considerable interference with the subject’s daily activities, unacceptable
- **Causality:**

| **Not related** | Relationship to the device, comparator or procedures can be excluded when:   - the event has no temporal relationship with the use of the investigational device, or the procedures related to application of the investigational device - the serious adverse event does not follow a known response pattern to the medical device (if the response pattern is previously known) and is biologically implausible; - the discontinuation of medical device application or the reduction of the level of activation/exposure - when clinically feasible - and reintroduction of its use (or increase of the level of activation/exposure), do not impact on the serious adverse event; - the event involves a body-site or an organ that cannot be affected by the device or procedure; - the serious adverse event can be attributed to another cause (e.g. an underlying or concurrent illness/ clinical condition, an effect of another device, drug, treatment or other risk factors); - the event does not depend on a false result given by the investigational device used for diagnosis, when applicable;   In order to establish the non-relatedness, not all the criteria listed above might be met at the same time, depending on the type of device/procedures and the serious adverse event. |
| --- | --- |
| **Possible** | The relationship with the use of the investigational device or comparator, or the relationship with procedures, is weak but cannot be ruled out completely. Alternative causes are also possible (e.g. an underlying or concurrent illness/ clinical condition or/and an effect of another device, drug or treatment). Cases where relatedness cannot be assessed, or no information has been obtained should also be classified as possible. |
| **Probable** | The relationship with the use of the investigational device or comparator, or the relationship with procedures, seems relevant and/or the event cannot be reasonably explained by another cause. |
| **Causal relationship** | The serious adverse event is associated with the investigational device, comparator or with procedures beyond reasonable doubt when:  - the event is a known side effect of the product category the device belongs to or of similar devices and procedures;   - the event has a temporal relationship with investigational device use/application or procedures; - the event involves a body-site or organ that   - - the investigational device or procedures are applied to;     - the investigational device or procedures have an effect on; - the serious adverse event follows a known response pattern to the medical device (if the response pattern is previously known); - the discontinuation of medical device application (or reduction of the level of activation/exposure) and reintroduction of its use (or increase of the level of activation/exposure), impact on the serious adverse event (when clinically feasible); - other possible causes (eg, an underlying or concurrent illness/clinical condition or/and an effect of another device, drug or treatment) have been adequately ruled out; - harm to the subject is due to error in use; - the event depends on a false result given by the investigational device used for diagnosis, when applicable;   In order to establish the relatedness, not all the criteria listed above might be met at the same time, depending on the type of device/procedures and the serious adverse event. |

### Timelines for reporting

- After informed consent has been obtained but prior to first use of the IMD, only adverse events caused by a study specific procedure should be reported
- After first use of the IMD, adverse events will be reported as follows:
  - All AEs, SAEs, AESIs and Device Deficiencies will be reported until 7 days after last use of IMD or until last follow-up visit (whichever occurs first)

All SAEs and AESI as defined in the protocol must be reported to the Sponsor within 24 hours of the trial staff becoming aware of the event. The immediate report shall be followed by detailed, written reports. The immediate and follow-up reports shall identify subjects by code numbers.

SAE details will be reported by the Investigator to the sponsor:

- By completing the SAE form in the (e)CRF

If an authorised Investigator from the reporting site is unavailable, initial reports without causality and expectedness assessment should be submitted to the Sponsor by a healthcare professional within 24 hours of becoming aware of the SAE, but must be followed-up by medical assessment as soon as possible thereafter.

### Follow-up

The Investigator must record follow-up information by updating the patient’s medical records and the appropriate forms in the (e)CRF. The worst case severity and seriousness of an event must be kept throughout the trial.

SAE follow-up information should only include new (e.g. corrections or additional) information and must be reported within 24 hours of the Investigator’s first knowledge of the information. This is also the case for previously non-serious AEs which subsequently become SAEs.

- All *SAEs* must be followed up until the outcome of the event is ‘recovered’, ‘recovered with sequelae’, ‘not recovered’ (in case of death due to another cause) or ‘death’ (due to the SAE) and until all related queries have been resolved, or until end of trial (whichever occurs first).
- *Non-serious AEs* must be followed up until the patient’s last study visit, and until all related queries have been resolved.

**SAEs after the end of the trial:** If the Investigator becomes aware of an SAE with suspected causal relationship to the IMD or experiment after the subject has ended the trial, the Investigator should report this SAE within the same timelines as for SAEs during the trial.

### Pregnancy

All participants in this study are pregnant subjects.

### Death

All deaths will be reported without delay to the sponsor (irrespective of whether the death is related to disease progression, the IMD, study procedure or is an unrelated event). The sponsor will notify all deaths as soon as possible after becoming aware to the EC and provide additional information if requested.

Fetuses with CDH are however at higher risk of in utero fetal death (around <5%). Also, the fetuses eligible for FETO are by definition at increased risk for neonatal death (>75%). Therefore condition related deaths, such as spontaneous intra-uterine fetal death, or neonatal death due to ventilatory insufficiency and pulmonary hypertension, will be reported but will not lead to immediate (temporary) suspension of the trial.

## Recording and reporting of Device Deficiencies

Each Device Deficiency must be documented by the Investigator in the source documents and reported to the Sponsor on a Device Deficiency form.

If the Device Deficiency leads to the occurrence of a (S)ADE, the (S)ADE must also be reported by the Investigator to the Sponsor on the appropriate forms and within the specified timelines.

## Reporting requirements to Ethics Committee’s (EC’s) and Competent Authorities (CA’s)

The Investigator is responsible for ensuring that all safety events are recorded in the (e)CRF and reported to the Sponsor in accordance with instructions provided below.

The Sponsor will promptly evaluate all SAEs, AESIs and Device Deficiencies against medical experience to identify and expeditiously communicate possible new safety findings to Investigators, ECs and applicable CA’s based on applicable legislation.

### Sponsor’s reporting of Serious Adverse Events and Device Deficiencies

The Sponsor is responsible to report to the CA’s where the clinical investigation has commenced:

- Any SAE that has a **causal** relationship with the investigational device, the comparator or the investigation procedure or where such causal relationship is reasonably possible;,
- Any Device Deficiency that might have led to a SAE if:

1. Appropriate action had not been taken or,
2. Intervention had not occurred or,
3. If circumstances had been less fortunate

- New findings/update in relation to already reportable events.

These ‘reportable events’ must be reported within the following timelines:

- A reportable event which results in imminent risk of death, serious injury, or serious illness that requires prompt remedial action for other patients/subjects, users or other persons or a new finding to it must be reported immediately, but not later than **2 calendar days** after awareness by the sponsor of a new reportable event or of new information in relation with an already reported event.
- Any other reportable event or a new finding/update to it must be reported immediately, but not later than **7 calendar days** following the date of awareness by the sponsor of the new reportable event or of new information in relation with an already reported event.

### Annual reporting

The Sponsor has the obligation to, once a year throughout the clinical trial (or on request), submit a progress report to the EC’s and CA’s containing an overview of all SADEs occurred during the reporting period and taking into account all new available safety information received during the reporting period.

### Overview reporting requirements

|  | WHAT | HOW | TO | TIMELINES |
| --- | --- | --- | --- | --- |
| Investigator | AE | AE form | sponsor | as defined in protocol |
|  | SAE | SAE form | sponsor | asap, but no later than 3 calendar days after awareness |
|  | Device Deficiency (DD) | DD form  + AE/SAE form  (if applicable) | sponsor | as defined in protocol  (exception: within 3 calender days if considered reportable event) |
|  | death | SAE form | sponsor | asap |
| Sponsor | all reportable events  (of all participating sites) | EU SAE report form (excel)^1^ | - Ethics Committees - CA for Belgium -> FAGG: via mail to [ct.rd@fagg.be](mailto:ct.rd@fagg.be) | asap, but no later than   - 2 calendar days (in case of risk of death or serious injury/illness that requires prompt remedial action for other patients, users or other persons) - 7 calendar days  (all other reportable events) |
|  | death | SAE form + narrative | - Ethics Committees | asap |
|  | Annual Progress Report | APR template | - Ethics Committees - CA for Belgium -> FAGG: via CESP portal | annually |

^1^ The SAE report form in excel format can be downloaded from the following web page: https://ec.europa.eu/health/sites/health/files/md_sector/docs/md_mdcg_2020-10-2_guidance_safety_report_form_en.xlsx?web=1

## Data Safety Monitoring Board (DSMB)

These following experts have agreed to participate in the DSMB:

| **Name** | **Affiliation** |
| --- | --- |
| **Francois Luks (Chair)** | Paediatric Surgeon-in-chief, Hasbro Children´s Hospital, Brown University, Providence, United States of America. |
| **Eduard Gratacos** | Fetal Medicine Specialist, experienced fetal surgeon, BCNatal, Barcelona Center for Maternal-Fetal and Neonatal Medicine, Hospital Clínic and Hospital Sant Joan de Deu), University of Barcelona, Spain. |
| **Tim Van Mieghem** | Fetal medicine specialist, experienced fetal surgeon, Ontario Fetal Centre, Division of Maternal Fetal Medicine, Mount Sinai Hospital, University of Toronto, Canada. |
| **Angélique Rézer** | Lawyer, Clinical Trial Centre, University Hospitals Leuven, Leuven, Belgium |
| **Frank Meijer** | Patient representative, Parent forum CHD. |

# Statistics and Data Analysis

Statistical analysis will be performed in accordance with ICH E9. ICH E3 and E8 will guide the structure and content of the clinical trial report.

## Sample Size Determination

We hypothesize that the balloon will deflate and will be expelled from the fetal airways in 100% of cases based on previously conducted studies. This needs to be confirmed in 23 patients in order to achieve a 95% confidence interval with a lower boundary of 85% (calculation of the interval confidence of a proportion using the *exact method*)[19], and using the website [www.sample-size.net](http://www.sample-size.net). With an expected loss rate of 8% (n=2) due to the need for removal on placental circulation (5%) or spontaneous balloon deflation (3), a total of 25 patients will need to be recruited. Based on a prudent estimate and current case load, the study should be finished within five years.

## Statistical Analysis

A descriptive statistical analysis of patient characteristics will be performed under the responsibility of Prof. Liesbeth Lewi. For all analyses, quantitative data will be expressed as median and inter-quartile-range (IQR), qualitative data will be expressed as numbers and percentages. The percentage of patients for whom the balloon has deflated according to the prescribed procedure and expelled from the fetal airways will be calculated with its 95% confidence interval (using the binomial method)[19].

The procedure's safety will be evaluated by describing the numbers and percentages of Adverse Events (AEs), Serious Adverse Events (SAEs) and Adverse Events of Special Interest (AESI). Other secondary endpoints (Prenatal: lung growth, spontaneous balloon deflation prior MRI exposure, localisation of the balloon within 24 hours following deflation by MRI, airway patency as on MRI following deflation, gestational age at membrane rupture; Postnatal: gestational age at delivery, balloon expulsion from the fetal airways, location of the balloon after delivery; Neonatal: Tracheal diameter on first postnatal chest X-ray; At discharge from the neonatal unit: assessment for any local side effects of the balloon (signs or symptoms of tracheomegaly and /or tracheomalacia), survival will be described in the study population.

No missing data is expected for the primary outcome. For secondary outcomes: since this is a descriptive study, we will not imputate missing data. Instance of missing values will be investigated individually to evaluate any potential reason why they are missing. A sensitivity analysis will be performed for missing data

A flowchart will describe the included patients who benefited from the Smart-TO balloon (and if not for what reason) and who performed the various stages of follow-up (Balloon placement, control US, removal of the balloon, birth and discharge from hospital).

### Analysis

| Endpoint | Statistical Analysis Methods |
| --- | --- |
| Primary | The percentage of patients for whom the balloon has deflated according to the prescribed procedure will be calculated with its 95% confidence interval (using the binomial method)[19] |
| Secondary | -Continuous measures will be expressed as median and IQR.  -Dichotomous measures will be expressed as number (%) |

### Other Analysis

There is no other analysis planned.

## Interim Analysis and Final Database Lock

There is no interim analysis.

# Data handling

Collected data will be coded and stored on a University Hospital firewall protected computer and secured servers, accessible via password for security and safety. Access to participant data will be restricted to clinical staff treating the participants. Each participant will be assigned with a study identification number before data collection from the hospital electronic database and case notes. Therefore, no names will be recorded.

The collection, processing and disclosure of personal data, such as patient health and medical information is subject to compliance with applicable personal data protection and the processing of personal data (REGULATION (EU) 2016/679 OF THE EUROPEAN PARLIAMENT AND OF THE COUNCIL of 27 April 2016 on the protection of natural persons with regard to the processing of personal data and on the free movement of such data, and repealing Directive 95/46/EC (General Data Protection Regulation).

The data manager of this study will be Dr Francesca Russo, MD PhD. Data will be managed and stored using the research-focused electronic data capture system (REDCap). REDCap is a secure, web-based applications designed exclusively to support data capture for research studies. REDCap is a PHP web application served by Apache Tomcat over a 128-bit SSL connection using a signed certificate. The application relies on a study-specific data dictionary defined in an iterative self-documenting process that will be conducted by the research team. The data dictionary is the foundation for custom case report form design and validated coding of variables. The REDCap database is on a secure server at KU Leuven with web-based data entry. The application is password protected and access will be only possible to the personnel involved in the study. Authentication of research staff will be assigned by the PI. The application generates a complete audit trail of user activity, provides reporting, and has an automated export mechanism to common statistical packages. Published results will not contain any personal data that could allow identification of individual participants.

## Data Collection Tools and Source Document Identification

### Operational aspects

Data collection, handling, processing and transfer for the purpose of this Trial will be performed in compliance with applicable regulations, guidelines for clinical trials and internal procedures, as follows:

#### Data collection

**Source Data** will be collected and recorded in the Trial participant’s files/medical records.

If applicable, worksheets may be used for capturing some specific data in order to facilitate completion of the (e)CRF. Any such worksheets will become part of the Trial participant's source documentation and will be filed together with or as part of the medical records (during but also following completion of the Trial).

It remains the responsibility of the Investigator to check that all data relating to the Trial, as specified in the Trial protocol, are entered into the (e)CRF in accordance with the instructions provided and that the forms are filled out accurately, completely and in a timely manner.

(e)CRFs are provided by the Sponsor for each participant. The Trial data will be transcribed from the source records (i.e. participant’s medical file or Trial-specific source data worksheets) into an (e)CRF by Trial Staff. Transcription to the (e)CRF will be done as soon as possible after a participant visit and in a pseudonymized manner using a unique identifier assigned by the Sponsor.

The (e)CRFs will be available for review at the next scheduled monitoring visit (as applicable) and shall under no circumstances capture personal data such as but not limited to the participant or their relative(s) name, home address, contact details, full date of birth medical record number (e.g. UZ Leuven EAD number), social security number etc.

#### Data Validation

All data relating to the Trial must be prepared and validated by the Investigator. Any (e)CRF entries, corrections and alterations must be made by the Investigator or other authorized Trial staff.

Proper audit trails must be available to demonstrate the validity of the Trial data collected. This includes historical records of original data entries, by whom and when the data was entered, as well as detailed records of any corrections or additions made to the original data entry (i.e. who made the correction/addition, when and why), without obliterating the original data entry information.

#### Data Management

The Trial Data Manager will perform extensive consistency checks on the received data. Queries will be issued in case of inconsistencies in accordance with internal procedures. A Data Management Plan (DMP) will be developed to map data flows, data validation measures that will be taken, how (interim) database lock(s) will be managed and, as applicable, the role and responsibilities of the Data Safety Monitoring Committee (DSMB)

#### Data Transfer

Any participant records or datasets that are transferred to the Sponsor or any partners of the Sponsor will contain the Trial-specific participant identifier only; participant names or any information which would make the participant identifiable will not be transferred. All pseudonymized data relating to the Trial must be transmitted in a secure manner to the Sponsor or any partners of the Sponsor(see 8.1.2. legal requirements).

### Legal requirements

All source data will be kept at a secured location with restricted access at all times. These data must be collected and processed with adequate precautions to ensure confidentiality and compliance with applicable data protection laws and regulations and more in particular the EU General Data Protection Regulation 2016/679 (GDPR) and relevant national laws implementing the GDPR. Appropriate technical and organizational measures to protect the data against unauthorized disclosure or access, accidental or unlawful destruction, or accidental loss or alteration must be established. Trial staff whose responsibilities require access to personal data agree to keep the data confidential.

The Investigator and the Participating Site(s) (as applicable) shall treat all information and data relating to the Trial disclosed to them as confidential and shall not disclose such information to any third parties or use such information for any purpose other than the objectives of the Trial as described in this protocol. The collection, processing and disclosure of personal data, such as participant health and medical information is subject to compliance with applicable laws and regulations regarding personal data protection and the processing of personal data.

The Investigator will maintain all source documents and completed (e)CRFs that support the data collected from each Trial participant, and will maintain a Trial Master File (TMF)/Investigator Site File (ISF) containing all Trial documents as specified in ICH-GCP E6(R2) Chapter 8 entitled “Essential Documents for the Conduct of a Clinical Trial”, and as specified by applicable regulatory requirement(s).
The Investigator will take appropriate measures to prevent accidental or premature destruction of these documents.

Transfer of the pseudonymized data will be performed via a secured method of transfer taking into account all applicable security arrangements and regulations (such as the European General Data Protection Regulation). The receiving party will be bound by contractual agreement to keep the transferred data confidential at all times and to only process the data for the purpose of the Trial. To this end, appropriate Data Transfer Agreements (DTAs) will be established.

## Audits and Inspections

The Investigator will permit direct access to Trial data and documents for the purpose of monitoring, audits and/or inspections by authorized entities such as but not limited to: the Sponsor or its designees and competent regulatory or health authorities. As such (e)CRFs, source records and other Trial related documentation (e.g. Investigator Site File, the Trial Master File, pharmacy records, etc.) must be kept current, complete and accurate at all times.

## Monitoring

In accordance with ICH-GCP E6(R2) the Sponsor is responsible for monitoring the Trial to ensure compliance with GCP and current legislation, and to verify, among other requirements, that proper written informed consent has been obtained and documented, that the Trial procedures have been followed as shown in the approved protocol, and that relevant Trial data have been collected and reported in a manner that assures data integrity. To this end Source Data will be compared with the data recorded in the (e)CRF. A risk-based approach will be applied to determine the extent of monitoring activities and monitoring of the Trial will be performed by qualified individuals (independent from the site Trial staff), as applicable. The Sponsor and Investigator/Participating Site will permit direct access to the Trial data and corresponding Source Data, and to any other Trial related documents or materials to verify the accuracy and completeness of the data collected.

## Archiving

As specified in ICH-GCP E6(R2) section 8.1 Addendum, the Sponsor and Investigator/Participating Site will maintain a record of the location(s) of all respective Essential Trial Documents (including but not limited to Source Documents, completed and final (e)CRF and ISF/TMF). The Sponsor should ensure that the Investigator has control of and continuous access to the (e)CRF data reported to the Sponsor during the Trial.

The Investigator/Participating Site should have control of all Essential Documents and records generated by the Investigator/Participating Site before, during and following termination of the Trial.

The Sponsor is responsible for archiving Trial specific documentation (such as but not limited to the Trial protocol, any amendments thereto, the final Clinical Study Report (CSR) and the Trial database) according to ICH-GCP E6(R2). Source data and site-specific Trial documents (such as but not limited to the original signed ICFs) will be archived by the participating site(s) according to local practice, and for at least 25 years following termination of the Trial. Archived data may be held on electronic record, provided that media back-up exists, hard copies can be obtained, if required and measures are taken to prevent accidental or premature loss or destruction of data. Destruction of Essential Documents prior to, during or upon completion of the required archival period, will require written authorisation from the Sponsor.

# Ethical and Regulatory Considerations

## Ethics Committee (EC) review & reports

Before the start of the Trial, this protocol and other related documents (e.g. ICF, advertisements, IB, etc.) will be submitted for review to the EC and to the relevant CA for Trial authorization. The Trial shall not commence until such approvals have been obtained.

It is the responsibility of the CI to produce the Annual Progress Report (APR) and submit to the EC/CA within 30 days of the anniversary date on which favourable opinion to start the Trial was given, and annually until the Trial is declared ended.

The CI shall notify the EC/CA of the end of the Trial. Should the Trial be temporarily suspended or, ended prematurely, the CI will notify the EC/CA and include the reasons for suspension/premature termination within 15 days of the decision. The CI will submit a final report with the results of the study, including any publications/abstracts, to the EC/CA within 1 year of trial termination or within 6 months for paediatric Trials.

## Peer review

This trial protocol did not undergo peer review.

## Regulatory Compliance

The trial will be conducted in compliance with the principles outlined in the requirements for the conduct of clinical trials in the EU as provided for in Directives 90/385/EEC and the Medical Device Regulation 2017/745 for the safety reporting, as well as in compliance with ICH-GCP E6(R2) guidelines, other GxP guidelines, ISO 14155:2011, the most recent version of the Declaration of Helsinki,  the Belgian law of May 7th 2004 regarding experiments on the human person (as amended) and with the EU General Data Protection Regulation 2016/679 (GDPR), the relevant Belgian laws implementing the GDPR, the Belgian Law of August 22^nd^ 2002 on patient rights and all other applicable legal and regulatory requirements.

## Protocol / GCP compliance

The Trial must be performed in accordance with the protocol, current ICH and ICH-GCP guidelines, and applicable regulatory and country-specific requirements. ICH guidelines are an international ethical and scientific quality standard for designing, conducting, recording, and reporting studies that involve the participation of human participants. Compliance with this standard provides public assurance that the rights, safety, and well-being of Trial participants are protected, consistent with the principles that originated in the most recent version of the Declaration of Helsinki, and that the Trial data are credible, reliable and reproducible.

The Investigator and Trial team acknowledge and agree that prospective, planned deviations or waivers to the protocol are not permitted under applicable regulations on clinical studies. However, should there be an accidental protocol deviation, such deviation shall be adequately documented in the source documents and on the relevant forms and reported to the CI and Sponsor. Deviations should also be reported to the EC as part of the EC’s continued review of the Trial (e.g. through the ASR, APR, etc.). Protocol deviations which are found to frequently recur, will require (immediate) action. The Investigator acknowledges that such recurring protocol deviations could potentially be classified as a serious violation of ICH and/or the protocol.

It is understood that “a serious violation” is likely to affect to a significant degree:

- the safety or physical or mental integrity of the Trial participants; or
- the scientific validity of the Trial

The Investigator is expected to take any immediate action required to protect the safety of any participant included in the Trial, even if this action represents a deviation from the protocol. In such cases, the Sponsor should be notified of this action and the EC at the Trial site should be informed according to local procedures and regulations.

## Data protection and participant confidentiality

The Trial will be conducted in compliance with the requirements of the EU General Data Protection Regulation 2016/679 (GDPR), the relevant Belgian laws implementing the GDPR including the Belgian Privacy Act of 30 July 2018 on the protection of privacy in relation to the processing of personal data. Any collection, processing and disclosure of personal data, such as participant health and medical information is subject to compliance with the aforementioned personal data protection laws (cfr. Data Processing Annex (DPA) in Appendix). In case personal data is transferred outside the European Economic Area, safeguards will be taken to ensure that appropriate protection travels with the data in accordance with the GDPR. (<https://ec.europa.eu/info/law/law-topic/data-protection/international-dimension-data-protection/rules-international-data-transfers_en#documents>)

Any personal data shall be treated as confidential at all times including during collection, handling and use or processing, and the personal data (including in any electronic format) shall be stored securely at all times and with all technical and organizational security measures that would be necessary for compliance with EU and national data protection legislation (whichever is more stringent). The Sponsor shall take appropriate measures to ensure the security of all personal data and guard against unauthorized access thereto or disclosure thereof or loss or destruction while in its custody.

## Insurance

The Participating Site, the Investigator and Sponsor shall have and maintain in full force and effect during the term of this Trial, and for a reasonable period following termination of the Trial, adequate insurance coverage for: (i) medical professional and/or medical malpractice liability, and (ii) general liability.

***For Belgian Participating Sites***

Art 29 of the Belgian Law relating to experiments on human persons dated May 7^th^, 2004 applies.

Prior to the start of the Trial, the Sponsor shall enter into an insurance contract in order to adequately cover Trial participants from Belgian sites in accordance with art. 29 of the said law.

## Amendments

Unless for urgent reasons as specified in ICH-GCP E6(R2) section 4.5.4, amendments must not be implemented prior to EC and/or CA review and/or approval, as applicable.
In accordance with the Belgian law of May 7^th^ 2004 regarding experiments on humans, the Sponsor may develop a non-substantial amendment at any time during the Trial. If a substantial amendment to the clinical Trial agreement or the documents that supported the original application for the clinical Trial authorisation is needed, the Sponsor must submit a valid substantial amendment to the Competent Authority (CA) for consideration, and to the EC for review and approval. The CA and/or EC will provide a response in accordance with timelines defined by applicable regulations. It is the Sponsor’s responsibility to assess whether an amendment is substantial or non-substantial for the purpose of submission to the CA and/or EC.

Amendments to the Trial are regarded as ‘substantial’ when they are likely to have a significant impact on the safety or physical or mental integrity of the clinical Trial participants, or the scientific value of the Trial.

<https://ec.europa.eu/health/sites/health/files/files/eudralex/vol-10/2010_c82_01/2010_c82_01_en.pdf>

## Post-Trial activities

If this trial is successful the Smart-TO device may go into production.

# Research Registration, Dissemination of Results and Publication Policy

The Declaration of Helsinki (latest version) and European and Belgian regulations require that every research Trial involving human participants be registered in a publicly accessible database before recruitment of the first participant. The CI is responsible for registering the Trial.

In addition, the CI will fulfil their ethical obligation to disseminate and make the research results publicly available. As such the CI is accountable for the timeliness, completeness and accuracy of the reports. Researchers, authors, Sponsors, editors and publishers must adhere to accepted guidelines for ethical reporting. Negative and inconclusive, as well as positive results must be published or otherwise made publicly available. Sources of funding, institutional affiliations and conflicts of interest must be declared in publication.

Publications will be coordinated by the CI. Authorship to publications will be determined in accordance with the requirements published by the International Committee of Medical Journal Editors and in accordance with the requirements of the respective medical journal.

For multicentre Trials, it is anticipated that the primary results of the overall Trial shall be published in a multicentre publication.

Participating Sites are not allowed to publish any subset data or results from the Trial prior to such multicentre publication.

Any publication by a Participating Site must be submitted to the Sponsor for review at least thirty (30) calendar days prior to submission or disclosure. Sponsor shall have the right to delay the projected publication for a period of up to three (3) months from the date of first submission to the Sponsor in order to enable the Sponsor to take steps to protect its intellectual property rights and know-how.

# Intellectual Property

Any know how, inventions, methods, developments, innovations, discoveries and therapies, whether patentable or not, arising from the Trial or made in the performance of the Trial protocol (“Inventions”) shall vest in the Sponsor. The Participating Site, its employees and Investigator(s) shall promptly disclose to the Sponsor any such Inventions. Parties have expressly agreed that any and all Trial data as collected and prepared in the performance of the Trial protocol shall be the sole property of Sponsor unless otherwise agreed in the clinical trial agreement.

# Joint Commission International (JCI)

In order to ensure the same quality and safety standards in patient care for clinical research as commonly applied by the Sponsor in its regular activities, and in accordance with JCI standards, the Sponsor shall comply with the following obligations: (a) the Sponsor will use trained and qualified employees or contractors to manage and coordinate the Trial; (b) the Sponsor will ensure that multi-center Trial reporting is reliable and valid, statistically accurate, ethical, and unbiased. (c) the Sponsor will not grant incentives, other than standard compensations and reimbursement of costs, to Trial participants or to participating site’s staff that would compromise the integrity of the research; (d) the Sponsor is responsible for monitoring and evaluating the quality, safety, and ethics of the Trial and will respect the participating site’s policies and processes when performing such monitoring and evaluation activities; (e) the Sponsor will protect the privacy and confidentiality of the Trial participants in accordance with all applicable laws.

# References

1. Snoek, K.G., et al., *Standardized Postnatal Management of Infants with Congenital Diaphragmatic Hernia in Europe: The CDH EURO Consortium Consensus - 2015 Update.* Neonatology, 2016. **110**(1): p. 66-74.

2. Ameis, D., N. Khoshgoo, and R. Keijzer, *Abnormal lung development in congenital diaphragmatic hernia.* Semin Pediatr Surg, 2017. **26**(3): p. 123-128.

3. Deprest, J.A., et al., *The making of fetal surgery.* Prenat Diagn, 2010. **30**(7): p. 653-67.

4. Jani, J.C., et al., *Severe diaphragmatic hernia treated by fetal endoscopic tracheal occlusion.* Ultrasound Obstet Gynecol, 2009. **34**(3): p. 304-10.

5. Russo, F.M., et al., *Fetoscopic endoluminal tracheal occlusion reverses the natural history of right-sided congenital diaphragmatic hernia: a European multicenter experience.* Ultrasound Obstet Gynecol, 2020.

6. Done, E., et al., *Predictors of neonatal morbidity in fetuses with severe isolated congenital diaphragmatic hernia undergoing fetoscopic tracheal occlusion.* Ultrasound Obstet Gynecol, 2013. **42**(1): p. 77-83.

7. Jimenez, J.A., et al., *Balloon removal after fetoscopic endoluminal tracheal occlusion for congenital diaphragmatic hernia.* Am J Obstet Gynecol, 2017. **217**(1): p. 78.e1-78.e11.

8. Sananes, N., et al., *Evaluation of a new balloon for fetal endoscopic tracheal occlusion in the nonhuman primate model.* Prenat Diagn, 2019.

9. Basurto, D., et al., *Safety and efficacy of the Smart Tracheal Occlusion device in the diaphragmatic hernia lamb model.* Ultrasound Obstet Gynecol, 2020.

10. Basurto, D., et al., *New device permitting non-invasive reversal of fetal endoscopic tracheal occlusion: ex-vivo and in-vivo study.* Ultrasound Obstet Gynecol, 2020. **56**(4): p. 522-531.

11. Jimenez, J.A., et al., *Balloon removal after fetoscopic endoluminal tracheal occlusion for congenital diaphragmatic hernia.* Am J Obstet Gynecol, 2017. **217**(1): p. 78 e1-78 e11.

12. Jani, J., et al., *Observed to expected lung area to head circumference ratio in the prediction of survival in fetuses with isolated diaphragmatic hernia.* Ultrasound Obstet Gynecol, 2007. **30**(1): p. 67-71.

13. Russo, F.M., et al., *Proposal for standardized prenatal ultrasound assessment of the fetus with congenital diaphragmatic hernia by the European reference network on rare inherited and congenital anomalies (ERNICA).* Prenat Diagn, 2018.

14. Jani, J., et al., *Tracheal diameter at birth in severe congenital diaphragmatic hernia treated by fetal endoscopic tracheal occlusion.* Prenat Diagn, 2011. **31**(7): p. 699-704.

15. George, A.T. and S. Motiwale, *Magnets, children and the bowel: a dangerous attraction?* World J Gastroenterol, 2012. **18**(38): p. 5324-8.

16. Thomson, M., et al., *Paediatric Gastrointestinal Endoscopy: European Society for Paediatric Gastroenterology Hepatology and Nutrition and European Society of Gastrointestinal Endoscopy Guidelines.* J Pediatr Gastroenterol Nutr, 2017. **64**(1): p. 133-153.

17. Van der Veeken, L., et al., *Fetoscopic endoluminal tracheal occlusion and reestablishment of fetal airways for congenital diaphragmatic hernia.* Gynecol Surg, 2018. **15**(1): p. 9.

18. Gallot, D., et al., *Prenatal detection and outcome of congenital diaphragmatic hernia: a French registry-based study.* Ultrasound Obstet Gynecol, 2007. **29**(3): p. 276-83.

19. Vollset, S.E., *Confidence intervals for a binomial proportion.* Stat Med, 1993. **12**(9): p. 809-24.

Appendices

# Appendix 1: Clinical trial protocol history

Complete the table below for each protocol amendment, provide a summary of the modifications made to the protocol while referencing the relevant protocol sections that were altered. Copy, insert and complete the table for each subsequent protocol amendment, while maintaining the completed table for previous protocol amendments.
If no protocol amendments have been issued: keep the amendment table, but do not complete.

| \| **Original CTP version:** \| 1.0 dated 07-04-2021 \| \| --- \| --- \| |
| --- | --- | --- |

| **Amendment #1:** | 2.0 dated 11-05-2021 |
| --- | --- |
| Modifications made / Reason for amendment: | |
| 5.2 | IMD Accountability included |
| 6 | Safety reporting updated. |

| **Amendment #2:** | 3.0 dated 23-06-2021 |
| --- | --- |
| Modifications made / Reason for amendment: | |
| 1 | Sentence added: “the following test have been completed using an identical version (V0) of the Smart-TO device intended for clinical purposes”. |
| 3.1 | Eligibility for FETO included.   1. Women carrying a fetus with isolated CDH eligible for FETO, i.e. with severe or moderate hypoplasia:    1. Left sided CDH with severe (i.e. quotient of observed-to-expected- lung-to-head ratio under 25%) or moderate pulmonary hypoplasia (observed-to-expected- lung-to-head ratios of 25 to 34.9% (any liver position), or 35 to 44.9% with intrathoracic liver herniation) [Jani et al UOG 2007].    2. Right sided CDH with severe pulmonary hypoplasia (quotient of observed-to-expected- lung-to-head ratio under 50%) [Russo FM et al UOG 2020]. |
| 9.3 | Regulatory compliance section adapted to current legislation. |
|  |  |
| **Amendment #3:** | 4.0 dated 30-06-2021 |
| 7.2 | Missing values clarification; interim analysis removed. |
| 7.3 | Interim analysis removed. |
|  |  |

# Appendix 2: Data Processing Annex (DPA)

Definitions:

- “Protocol” means the document entitled *“Smart Tracheal Occlusion for Congenital Diaphragmatic Hernia: A Feasibility Study”* containing the details of the academic Trial as developed by the Sponsor and approved by the relevant Ethics Committee.
- “Sponsor” means University Hospitals Leuven (UZ Leuven).
- Participating site acts as a data processor as defined under article 4, 8) of the Regulation (EU) 2016/679 (“Data Processor”) for the Sponsor who acts as data controller as defined under article 4, 7) of the Regulation (EU) 2016/679 (“Data Controller”).
- “Applicable Law” means any applicable data protection or privacy laws, including:
  1. the Regulation (EU) 2016/679 also referred as the General Data Protection Regulation ("GDPR");
  2. other applicable laws that are similar or equivalent to or that are intended to or implement the laws that are identified in (a) of this definition;
- "Personal Data" means any information relating to an identified or identifiable natural person (”Data Participant”), including without limitation pseudonymized information, as defined in Applicable Law and described in the Protocol.

Rights and obligations:

1. The Data Processor is instructed to process the Personal Data for the term of the Trial and only for the purposes of providing the data processing tasks set out in the Protocol. The Data Processor may not process or use Personal Data for any purpose other than a Data Participant’s medical records, or other than provided in the instructions of the Trial protocol, including with regard to transfers of personal data to a third country or an international organization, unless the Data Processor is required to do so according to Union or Member State law.
2. Data Processor shall at all times maintain a record of processing of Personal Data in accordance with Applicable Law and if the Data Processor considers an instruction from the Data Controller to be in violation of the Applicable Law, the Data Processor shall promptly inform the Data Controller in writing about this.
3. The Data Processor must ensure that persons authorized to process the Personal Data have committed themselves to confidentiality or are under an appropriate statutory obligation of confidentiality.
4. The Data Processor shall implement appropriate technical and organizational measures to prevent that the Personal Data processed is:
5. accidentally or unlawfully destroyed, lost or altered,
6. disclosed or made available without authorization, or
7. otherwise processed in violation of Applicable Law.
8. The appropriate technical and organizational security measures must be determined with due regard for:
9. the current state of the art,
10. the cost of their implementation, and
11. the nature, scope, context and purposes of processing as well as the risk of varying likelihood and severity for the rights and freedoms of natural persons.
12. Taking into account the nature of the processing, the Data Processor shall assist the Data Controller, by means of appropriate technical and organizational measures, insofar as this is possible, in fulfilling its obligation to respond to requests from Data Participants pursuant to laws and regulations in the area of privacy and data protection (such as, the right of access, the right to rectification, the right to erasure, the right to restrict the processing, the right to data portability and the right to object)
13. The Data Processor shall upon request provide the Data Controller with sufficient information to enable the Data Controller to ensure that the Data Processor's obligations under this DPA are complied with, including ensuring that the appropriate technical and organizational security measures have been implemented.
14. The Data Controller is entitled to appoint at its own cost an independent expert, reasonably acceptable to the Data Processor, who shall have access to the Data Processor's data processing facilities and receive the necessary information for the sole purpose of auditing whether the Data Processor has implemented and maintained said technical and organizational security measures. The expert shall upon the Data Processor's request sign a non-disclosure agreement provided by the Data Processor, and treat all information obtained or received from the Data Processor confidentially, and may only pass on, after conferral with the Data Processor, the findings as described under 10) (ii) below to the Data Controller.
15. The Data Processor must give authorities who by Union or Member State law have a right to enter the Data Controller's or the Data Controller's processors’ facilities, or representatives of the authorities, access to the Data Processor's physical facilities against proper proof of identity and mandate, during normal business hours and upon reasonable prior written notice.
16. The Data Processor must without undue delay in writing notify the Data Controller about:
17. any request for disclosure of Personal Data processed under the Protocol by authorities, unless expressly prohibited under Union or Member State law,
18. any finding of (a) breach of security that results in accidental or unlawful destruction, loss, alteration, unauthorized disclosure of, or access to, Personal Data transmitted, stored or otherwise processed by the Data Processor under the Protocol, or (b) other failure to comply with the Data Processor's obligations, or
19. any request for access to the Personal Data (with the exception of medical records for which the Data Processor is considered data controller) received directly from the Data Participants or from third parties.
20. Such a notification from the Data Processor to the Data Controller with regard to a breach of security as meant in 10) (ii)(a) above will contain at least the following information:
21. the nature of the Personal Data breach, stating the categories and (by approximation) the number of Data Participants concerned, and stating the categories and (by approximation) the number of the personal data registers affected (datasets);
22. the likely consequences of the Personal Data breach;
23. a proposal for measures to be taken to address the Personal Data breach, including (where appropriate) measures to mitigate any possible adverse effects of such breach.
24. The Data Processor shall document (and shall keep such documentation available for the Data Controller) any Personal Data breaches, including the facts related to the Personal Data breach, its effects and the corrective measures taken. After consulting with the Data Controller, the Data Processor shall take any measures needed to limit the (possible) adverse effects of Personal Data breaches (unless such consultation cannot be awaited due to the nature of the Personal Data breach).
25. The Data Processor must promptly and reasonably assist the Data Controller (with the handling of (a) responses to any breach of security as described in 10) (ii) above and (b) any requests from Data Participants under Chapter III of the GDPR, including requests for access, rectification, blocking or deletion. The Data Processor must also reasonably assist the Data Controller by implementing appropriate technical and organizational measures for the fulfilment of the Data Controller's obligation to respond to such requests.
26. The Data Processor must reasonably assist the Data Controller with meeting the other obligations that may be incumbent on the Data Controller according to Union or Member State law where the assistance of the Data Processor is implied, and where the assistance of the Data Processor is necessary for the Data Controller to comply with its obligations. This includes, but is not limited to, at the request to provide the Data Controller with all necessary information about an incident under 10) (ii), and all necessary information for an impact assessment in accordance with Article 35 and Article 36 of the GDPR.

Subprocessor:

1. The Data Processor may only engage a subprocessor, with prior specific or general written consent from the Data Controller. The Data Processor undertakes to inform the Data Controller of any intended changes concerning the addition or replacement of a subprocessor by providing a reasonable prior written notice to the Data Controller. The Data Controller may reasonably and in a duly substantiated manner object to the use of a subprocessor. The Data Processor must inform the Data Controller in writing of the discontinued use of a subprocessor.
2. Prior to the engagement of a subprocessor, the Data Processor shall conclude a written agreement with the subprocessor, in which at least the same data protection obligations as set out in this DPA shall be imposed on the subprocessor, including obligations to implement appropriate technical and organizational measures and to ensure that the transfer of Personal Data is done in such a manner that the processing will meet the requirements of the Applicable Law.
3. The Data Controller has the right to receive a copy of the relevant provisions of Data Processor's agreement with the subprocessor related to data protection obligations. The Data Processor shall remain fully liable to the Data Controller for the performance of the subprocessor obligations under this DPA. The fact that the Data Controller has given consent to the Data Processor's use of a subprocessor is without prejudice for the Data Processor's duty to comply with this DPA.

# Appendix 3: Figures

**Figure 1:** (A) Smart-TO components; (B) the Smart-TO device in its package; (C) inflation catheter; (D) stylet; (E) the Smart-TO device in its deflated state; (F) the Smart-TO device in its inflated state.


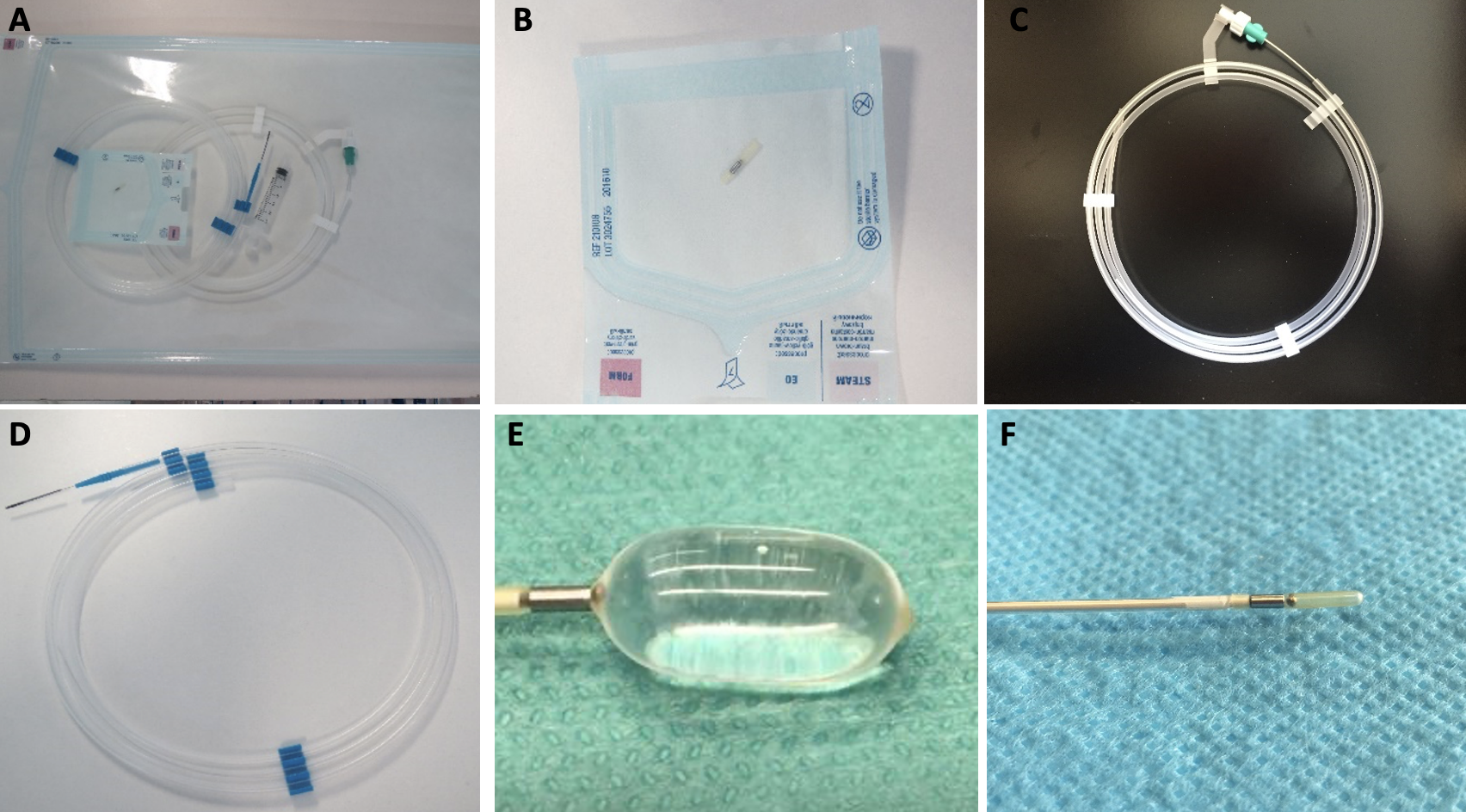


**Figure 2**: (A) The Smart-TO device mounted on the fetoscope; (B) Fetoscopic endoluminal tracheal occlusion (FETO) prcocedure.


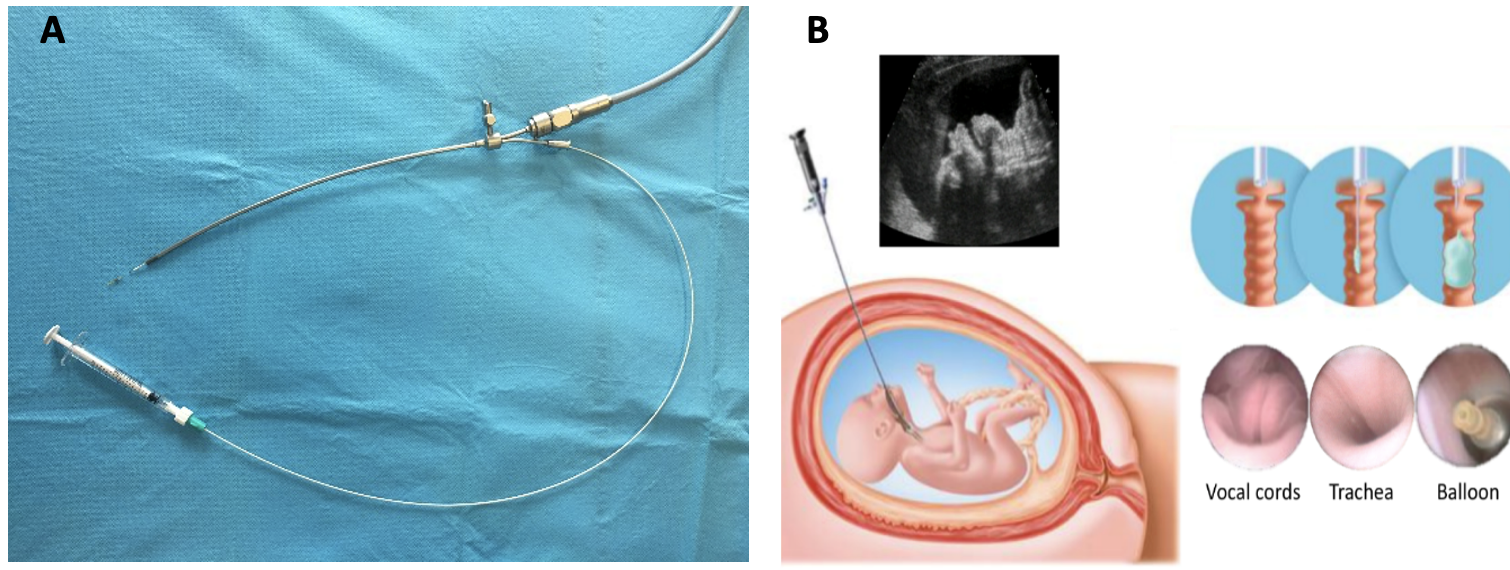


**Figure 3**: Smart-TO device Instructions of use


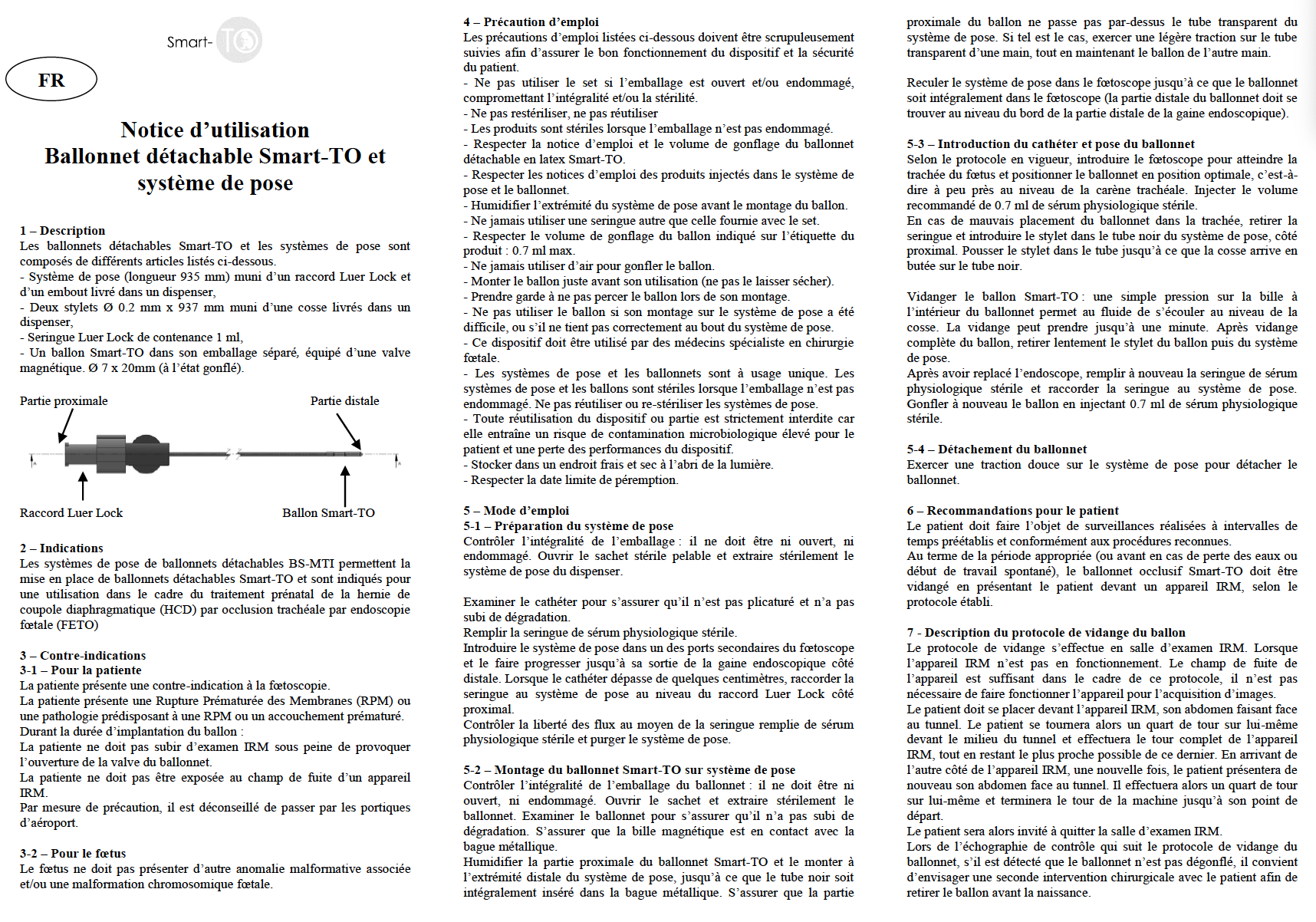


**Figure 4**: Description of the movements to be performed around the MRI (Pregnant patient seen from above).


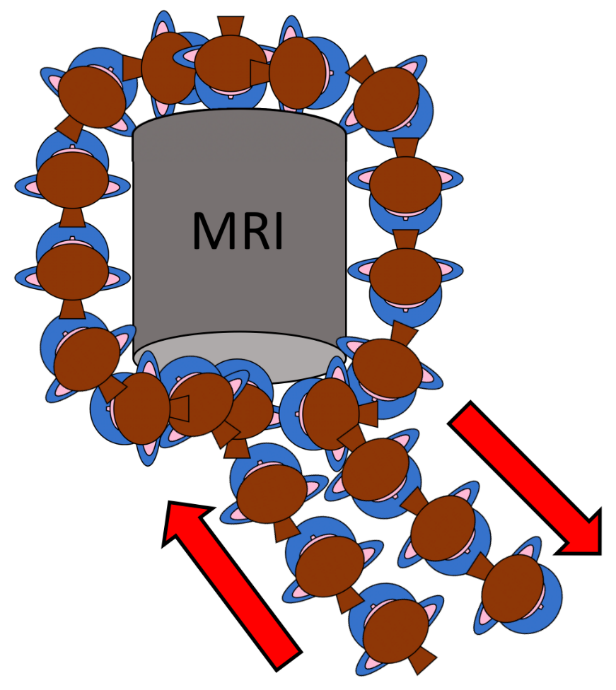

Supplement: S1 Text — (DOCX) [file pone.0273878.s003.docx]
